# Genome-wide association study followed by trans-ancestry meta-analysis identify 17 new risk loci for schizophrenia

**DOI:** 10.1186/s12916-021-02039-9

**Published:** 2021-08-12

**Authors:** Jiewei Liu, Shiwu Li, Xiaoyan Li, Wenqiang Li, Yongfeng Yang, Suqin Guo, Luxian Lv, Xiao Xiao, Yong-Gang Yao, Fanglin Guan, Ming Li, Xiong-Jian Luo

**Affiliations:** 1 Key Laboratory of Animal Models and Human Disease Mechanisms of the Chinese Academy of Sciences & Yunnan Province, Kunming Institute of Zoology, Chinese Academy of Sciences, Kunming, 650223 Yunnan China; 2 Kunming College of Life Science, University of Chinese Academy of Sciences, Kunming, 650204 Yunnan China; 3 Henan Mental Hospital, The Second Affiliated Hospital of Xinxiang Medical University, Xinxiang, 453002 Henan China; 4 Henan Key Lab of Biological Psychiatry, International Joint Research Laboratory for Psychiatry and Neuroscience of Henan, Xinxiang Medical University, Xinxiang, 453002 Henan China; 5 KIZ-CUHK Joint Laboratory of Bioresources and Molecular Research in Common Diseases, Kunming Institute of Zoology, Chinese Academy of Sciences, Kunming, 650223 Yunnan China; 6 CAS Center for Excellence in Brain Science and Intelligence Technology, Chinese Academy of Sciences, Shanghai, 200031 China; 7 Department of Forensic Psychiatry, School of Medicine & Forensics, Xi’an Jiaotong University Health Science Center, Xi’an, 710061 Shaanxi China; 8 Center for Excellence in Animal Evolution and Genetics, Chinese Academy of Sciences, Kunming, 650223 China

**Keywords:** Schizophrenia, GWAS, Han Chinese, PRS, TWAS, Meta-analysis

## Abstract

**Background:**

Over 200 schizophrenia risk loci have been identified by genome-wide association studies (GWASs). However, the majority of risk loci were identified in populations of European ancestry (EUR), potentially missing important biological insights. It is important to perform 5 GWASs in non-European populations.

**Methods:**

To identify novel schizophrenia risk loci, we conducted a GWAS in Han Chinese population (3493 cases and 4709 controls). We then performed a large-scale meta-analysis (a total of 143,438 subjects) through combining our results with previous GWASs conducted in EAS and EUR. In addition, we also carried out comprehensive post-GWAS analysis, including heritability partitioning, enrichment of schizophrenia associations in tissues and cell types, trancscriptome-wide association study (TWAS), expression quantitative trait loci (eQTL) and differential expression analysis.

**Results:**

We identified two new schizophrenia risk loci, including associations in *SHISA9* (rs7192086, *P* = 4.92 × 10^-08^) and *PES1* (rs57016637, *P* = 2.33 × 10^−11^) in Han Chinese population. A fixed-effect meta-analysis (a total of 143,438 subjects) with summary statistics from EAS and EUR identifies 15 novel genome-wide significant risk loci. Heritability partitioning with linkage disequilibrium score regression (LDSC) reveals a significant enrichment of schizophrenia heritability in conserved genomic regions, promoters, and enhancers. Tissue and cell-type enrichment analyses show that schizophrenia associations are significantly enriched in human brain tissues and several types of neurons, including cerebellum neurons, telencephalon inhibitory, and excitatory neurons. Polygenic risk score profiling reveals that GWAS summary statistics from trans-ancestry meta-analysis (EAS + EUR) improves prediction performance in predicting the case/control status of our sample. Finally, transcriptome-wide association study (TWAS) identifies risk genes whose cis-regulated expression change may have a role in schizophrenia.

**Conclusions:**

Our study identifies 17 novel schizophrenia risk loci and highlights the importance and necessity of conducting genetic study in different populations. These findings not only provide new insights into genetic etiology of schizophrenia, but also facilitate to delineate the pathophysiology of schizophrenia and develop new therapeutic targets.

**Supplementary Information:**

The online version contains supplementary material available at 10.1186/s12916-021-02039-9.

## Background

Schizophrenia (SCZ) is a devastating mental disorder that affects about 0.5–1% of the world’s population [1]. The main symptoms of SCZ include positive symptoms (hallucinations and delusions), negative symptoms (anhedonia, alogia, and avolition), and cognitive impairments (impaired working memory and executive function) [2]. Due to the high mortality and considerable morbidity [3], SCZ imposes substantial economic burden on society and becomes a major threat to global health [4]. The pathophysiology of SCZ remains largely unknown. Nevertheless, lines of evidence indicate that SCZ has a strong genetic component. The heritability of SCZ was estimated around 0.80 [5], implying the major role of the inherited variants in SCZ. To dissect the genetic basis of SCZ, great efforts have been made and significant progresses have been achieved. Low-frequency variants such as structural variants [6], copy number variations [7–10], rare [11], and de novo mutations [12–16] were reported to be associated with SCZ. In addition, GWASs have identified over 200 risk loci that showed robust associations with SCZ [17–27].

Although recent large-scale studies have provided important insights into the genetic etiology of SCZ, challenges remain in dissecting the genetic architecture of SCZ. First, the majority of risk loci were identified in populations of European ancestry [21, 26]. Considering the diverse differences of allelic frequency and linkage disequilibrium pattern in different continental populations [28], performing GWAS in non-European populations will provide new insights into genetic etiology of SCZ. Second, despite the fact that a recent GWAS meta-analysis in populations of East Asian ancestry (EAS) revealed comparative genetic architecture of SCZ between populations of European ancestry (EUR) and East Asian ancestry (EAS) (genetic correlation between EAS and EUR is 0.98 ± 0.03), this study also showed population-specific associations [24]. For example, Lam et al. found that a large proportion of genome-wide significant variants identified in EAS showed dramatic differences in allelic frequency between EAS and EUR [24], further indicating the importance of conducting GWAS in non-European populations. Third, accumulating data suggest that a large proportion of risk variants contribute to SCZ through modulating gene expression [29–31]. Therefore, it is important to pinpoint the potential target genes of the identified risk variants. To address these challenges, we firstly conducted a GWAS in Han Chinese population (*N* = 8202). We then performed a large-scale meta-analysis (a total of 143,438 subjects) through combining our results with summary statistics from previous GWASs conducted in EAS and EUR (i.e., summary statistics-based meta-analysis, fixed-effect model was used) [24]. We also performed a transcriptome-wide association study (TWAS) to pinpoint the potential target genes of the identified risk variants and explored the potential tissue and cell type that the identified risk variants and genes may exert their biological effects.

## Methods

### Study subjects

SCZ cases were from inpatient and outpatient services of collaborating mental health centers of China. Part of cases has been described in our previous studies for candidate gene analyses [32–34]. Diagnosis was based on Diagnostic and Statistical Manual of mental disorders (DSM-IV) criteria, with the use of Structured Clinical Interview for DSM-IV (SCID) Axis I Disorders. All relevant and detailed information (including onset of SCZ, first onset or remitted, symptoms and chief complaint, duration course, family history of psychiatric disorders, medication history) were carefully evaluated by at least two independent experienced psychiatrists to reach a consensus DSM-IV diagnosis. Detailed information about diagnosis had been described in previous studies [33, 34]**.** The average age of cases and controls were 35.67 ± 10.29 and 28.82 ± 6.83 years, respectively. 37.45% cases and 54.53% controls were males, respectively. All participants provided written informed consents. This study was approved by the Ethical Committee and internal review board of the Kunming Institute of Zoology (No: SMKX-20191215-07) and participating hospitals and universities (including the Second Affiliated Hospital of Xinxiang Medical University and Xi’an Jiaotong University). The samples were recruited from 2010 to 2018.

### DNA extraction and genotyping

Genomic DNA was extracted from the peripheral blood with the use of QIAamp DNA blood mini kit (Cat. No: 51106). We used two types of genotyping platforms (arrays), including Illumina ASA (BeadChip Array Asian Screening Array-24+v1.0 HTS ASAMD-24v1-0) and GSA (BeadChip Array Global Screening Array-24+v2.0 HTS GSA v2.0+Multi-Disease). For ASA array (including 743,722 variants), 56.7% of the variants are common variants (with minor allele frequency > 0.05), 30.8% are low-frequency variant (with minor allele frequency between 0.01 and 0.05), and 12.5% are rare variants (minor allele frequency < 0.01). ASA array includes a broad spectrum of pharmacogenomics markers (*N* = 5588) obtained from CPIC guidelines (www.cpicpgx.org) and the PharmGKB database (www.pharmgkb.org). In addition, the ASA array contains about 50,000 SNPs selected from ClinVar database (www.ncbi.nlm.nih.gov/clinvar). For GSA array (*N* = 759,993 variants), 54.4% of the variants are common variants (with minor allele frequency > 0.05), 18.1% are low-frequency variant (with minor allele frequency between 0.01 and 0.05), and 27.4% are rare variants (minor allele frequency < 0.01). Similar with ASA array, the GSA array includes multiple expert curated variants obtained from ClinVar (www.ncbi.nlm.nih.gov/clinvar), PharmGKB (www.pharmgkb.org), NHGRI (www.genome.gov/), and other databases. More details about the ASA and GSA arrays can be found in the official Illumina website (https://www.illumina.com/products/by-type/microarray-kits). Genotyping assays were conducted at Guoke Biotechnology Co., LTD in Beijing (www.bioguoke.com).

### Quality control

We conducted quality control as previously described [23, 24, 35], with some minor revisions. The main consideration is to include more subjects and variants under the premise of ensuring data quality. The individual-level quality control (QC) was processed as follows: (1) Samples with genotype missing rate > 0.03 were excluded; (2) Samples with a heterozygosity (calculated by plink 1.9 –het command) deviated ± 3 stand deviation (s.d.) from the mean heterozygosity of all samples were excluded; (3) Related samples were inferred by KING software (http://people.virginia.edu/~wc9c/KING/) [36]. We used –related command implemented in KING to infer the potential kinship coefficients with 3rd degree. After inferring relatedness between samples, KING program exported related and unrelated samples. Related samples were then excluded. (4) We checked the sex of samples by using Plink (v1.09) (with --check-sex command). Samples with inconsistent sex information were excluded. We excluded samples if their sex estimated by genotype data and medical record information were inconsistent. In addition, we also excluded samples whose sex could not be accurately predicted based on the genotype data.

The SNP-level QC are as follows: (1) Excluding SNPs with a call rate < 97%; (2) As described in the study of Lam et al. [24], we excluded SNPs with a significant deviation from Hardy-Weinberg equilibrium (*P* < 1.0 × 10^−6^ in controls and *P* < 1.0 × 10^−10^ in cases); (3) Excluding SNPs with a minor allele frequency (MAF) < 0.01; (4) Only biallelic SNPs were retained for further analysis. The QCs were performed by Plink 1.9 software [37]. Our analysis started in March 2020 and finished in May 2021.

### Principal component analysis (PCA)

We performed principal component analysis (PCA) to assess population stratification and exclude the outliers. The subjects from the 1000 Genomes project (including CHB (Han Chinese in Beijing, China), CHS (Southern Han Chinese), JPT (Japanese in Tokyo, Japan), YRI (Yoruba in Ibadan, Nigeria), and CEU (Utah residents with northern and western European ancestry)) [38] were used as references and PCA was performed with GCTA software [39]. Samples that were not clustered with subjects of Han Chinese ancestry were excluded. We then used the Smartpca program to calculate principal components as covariates to correct potential population stratification [40, 41]. We excluded the MHC region (chr6: 25-34 MB) when performing PCA. Five PCA iterations were run and top 20 principal components (PCs) were calculated. Samples that were away from 6 standard deviations (s.d.) of the mean of each PC were excluded. After stringent quality control, 3493 cases and 4709 controls were retained for GWAS.

For each group (ASA subgroup 1 and subgroup 2, GSA), the top 20 PCs were calculated. The PCs were calculated as covariates to correct potential population stratification for each group (ASA subgroup 1 and subgroup 2, GSA), respectively. As suggested by Price et al. [41], we included a variable number of PCs as covariates to perform logistic regression analysis in each group (ASA subgroup1, ASA subgroup2, GSA). We used genomic control inflation factor (λ_GC_) to estimate the effect of the number of PCs on population stratification adjustment [41, 42]. The selection of the final number of PCs was mainly based on the following criteria [43]: (1) the λ_GC_ should as close to 1 as possible (indicating population stratification is well controlled); (2) when including more PCs, the λ_GC_ did not change significantly (indicating that the number of PCs selected is enough to adjust population stratification). Based on these criteria, we finally selected 10 PCs for ASA subgroup 1, 4 PCs for ASA subgroup 2, and 12 PCs for GSA samples respectively.

### Genotype imputation

The imputation of the genotypes was performed by Eagle [44] and minimac3 [45]. The Eagle was used for phasing the genotype data of each chromosome. The reference panel from the 1000 Genomes project (Phase 3) [38] was downloaded from the minimac3 website (https://genome.sph.umich.edu/wiki/Minimac3). The imputed data were further processed with the following QC steps: (1) SNPs with an imputation quality score < 0.8 were excluded; (2) SNPs with a MAF < 0.01 were excluded; (3) SNPs that significantly deviated from the Hardy-Weinberg equilibrium (*P* < 1 × 10^−6^ in controls, *P* < 1 × 10^−10^ in cases) were excluded; (4) Only SNPs with a genotype imputation rate > 97% were remained; (5) Only biallelic SNPs were included for further association analysis. After QC, the total number of SNPs included in GWAS analysis was 4,724,225 (for ASA arrays) and 5,107,135 (for GSA arrays). The number of overlapping SNPs between the two platforms is 3,937,527. To make our QC procedures more clear and easy to follow, we provided the detailed information about QC (to list all pre- and post-imputation QC steps with excluded and remaining SNPs and participants after each step) in Additional file 1: Figure S1.

### GWAS and meta-analysis

As our samples were genotyped with Illumina ASA and GSA Chip arrays, to avoid potential effects of different array platforms on our analysis, we performed genome-wide association analysis separately. We firstly performed genetic association analysis in our samples (ASA subgroup 1, ASA subgroup 2, GSA, respectively) by using logistic regression. This association analysis was based on genotype data of each group (i.e., ASA subgroup 1, ASA subgroup 2, GSA), with PCs included as covariate. The results from these three genome-wide summary statistics were then meta-analyzed (summary statistics-based meta-analysis, fixed-effect model was used). GWAS were performed by Plink (v1.09) software [37]. We further performed a summary statistics-based meta-analysis in East Asian populations through combining the results of our study and GWAS summary statistics (only EAS were used) from a recent study by Lam et al. (including 22,778 case and 35,362 controls) [24]. Finally, we carried out a trans-ancestry meta-analysis (summary statistics-based, fixed effect model) through combining our results with the summary statistics from East Asians and Europeans (containing 56,418 cases and 78,818 controls) [24, 26].

### Defining of independent risk loci and new loci identification

To identify independent risk loci, we used FUMA [46] to clump the association results (with the default parameters). Different reference panels were used for clumping. For the meta-analysis in EAS, subjects of East Asian ancestry from the 1000 Genomes project [38] were used. For the meta-analysis of EAS and PGC2 [24], subjects of European ancestry from the 1000 Genomes project [38] were used. The clumping processes were as follows: The minimum r^2^ threshold for independent significant SNPs was set to 0.6, which was used to define the boundaries of the genomic risk locus. When independent significant SNPs were defined in a locus, these SNPs were further processed to identify the lead SNP for each locus. The minimum r^2^ for defining lead SNPs was set to r^2^ = 0.1. The LD blocks of independent significant SNPs that < 250 kb were merged into a single genome locus. More details about LD clump can be found in FUMA website (https://fuma.ctglab.nl/tutorial). Visualization of association results of interest SNPs and its nearby variants were generated with locuszoom [47] (http://locuszoom.org/genform.php?type=yourdata).

We compared the genome-wide significant (GWS) loci identified in this study and published SCZ GWAS [21, 23, 24, 26], and we also included GWASs listed in GWAS catalog database in FUMA [46]. We used following approaches for risk loci comparison: (1) If the published SCZ GWAS provide GWS index SNPs and its corresponding genomic region (chromosomal coordinates), we overlapped genomic region of our GWS loci with these published risk loci. Non-overlapping loci were defined as new loci; (2) If the published SCZ GWAS only list GWS index SNPs, the newly identified risk loci (by us) should have no overlap with these GWS index SNPs, the index SNPs of the newly identified risk loci (by us) should also not be in linkage disequilibrium with the reported GWS index SNPs (R^2^ < 0.1); (3) GWAS catalog database implemented in FUMA [46] were also used to identify new SCZ GWS loci.

### Polygenetic risk score profiling

Polygenetic risk score profiling was performed by PRSice2 [48]. The PRS scores derived from training datasets were used to predict the case-control status of our samples. And the samples included in this study were used as target sample. We used classic clumping and threshold method. We run PRS using default parameters in PRSice2 [48]. The detailed clumping parameters of PRS calculation were as follows: --clump-kb 250, --clump-p 1.0, --clump-r2 0.10. Summary statistics from EAS [24], PGC2 EUR [24], EAS + EUR [24], and CLOZUK+PGC2 [21] were used as training datasets in PRS analysis. And we set 10 *P* value thresholds (5 × 10^−8^, 5 × 10^−5^, 0.0001, 0.001, 0.01, 0.05, 0.1, 0.2, 0.5, 1) to analyze the phenotypic variance explained at different *P* value cutoffs.

### Tissue and cell-type enrichment analysis

To explore if schizophrenia associations were enriched in specific human tissues, we performed tissue enrichment analysis by using MAGMA (Version 1.08) [49], which is implemented in FUMA software (Version 1.3.6a) [46]. Briefly, gene expression data of different human tissues (RNA sequencing data from the Genotype-Tissue Expression (GTEx) [50] consortium) were used to identify the genes that differentially expressed in a specific tissue. Based on the GWAS *P* values, MAGMA quantifies the degree of association between a gene and schizophrenia (i.e., obtain a gene-level *P* value) by using a multiple linear principal component regression model. MAGMA then tests if schizophrenia associations were enriched in the specifically expressed genes in a specific tissue. More detailed information about tissue enrichment analysis can be found in FUMA website (https://fuma.ctglab.nl/).

Cell-type enrichment analysis was also performed using MAGMA (Version 1.08) [49]. Single-cell RNA sequencing data from the mouse central nervous system (CNS) [51] were downloaded and processed as described in Bryois et al. [52]. Briefly, a total of 160,769 high-quality single-cell RNA-seq data were analyzed. Human genes were mapped to orthologous mouse genes (one to one) based on MGI annotations (http://www.informatics.jax.org/homology.shtml), and genes that were not expressed in the mouse central nervous system (CNS) were excluded. The expression specificity of a specific gene was calculated as described in Bryois et al. [52] and the top 10% that were most specifically expressed genes in each cell type were used for enrichment analysis. The *P* values of MAGMA enrichment analysis were corrected by false discovery rate (FDR).

### Gene set enrichment analysis

Gene set analysis was performed with MAGMA software (Version 1.08) [49]. MAGMA gene set analysis includes two main procedures. Firstly, by using the GWAS summary statistics and gene annotation files (NCBI b37, downloaded from official MAGMA website (https://ctg.cncr.nl/software/magma)), MAGMA maps SNPs to genes (with gene boundaries setting parameters “--annotate window = 35,10”). MAGMA then calculates the association strength between a specific gene and schizophrenia. A gene and phenotype association matrix was generated in this step with default “snp-wise = mean” model. The MHC region (from 25 to 34 Mb) on chromosome 6 was excluded in analysis. Secondly, gene set analysis which was also known as competitive gene set analysis was performed. MAGMA tests whether the association of a target gene set with phenotype is greater than other genes that are not included in the gene set. The *P* value of competitive gene set was used to determine the significance level. We downloaded gene sets from MSigDB database (v7.1) (http://software.broadinstitute.org/gsea/msigdb/) [53] and all GO terms (including cellular component, biological processes, and molecular functions). KEGG pathway gene sets were also included and a total of 10,378 gene set terms were compiled. We further retained 6194 gene sets with a gene number range from 10 to 200 for the final analysis. The final *P* values (i.e., the association strength between gene sets and schizophrenia) of MAGMA competitive gene sets were corrected by false discovery rate (FDR).

### LD score regression analysis

We conducted stratified linkage disequilibrium score regression (LDSC) [54] to partition SCZ SNP heritability and test enrichment of SCZ heritability in different functional annotations [54]. In addition, we also performed genetic correlation analysis between our Chinses cohort and the reported EAS samples (based on summary statistics) using LDSC [54].

### Transcriptome-wide association analysis

We performed TWAS using the summary statistics from all combined samples (including our Han Chinese cohort, EAS and PGC2 [24], a total of 59,911 cases and 83,527 controls) and brain eQTL (gene expression SNP weights) from the CommonMind Consortium (CMC, *N* = 452) [55]. CMC measured gene expression in the dorsolateral prefrontal cortex (DLPFC) of human brain with the use of RNA sequencing. The CMC gene expression SNP weights were derived from the FUSION pipeline (http://gusevlab.org/projects/fusion/). Detailed information about sample collection, RNA extraction and sequencing, genotyping and statistical analysis of CMC dataset can be found in the original studies [55]. TWAS was performed on autosomal chromosomes using the FUSION.assoc.test.R script across all predictive models, such as LASSO, GBLUP, Elastic Net, and BSLMM. To determine which is the best model for TWAS, FUSION performed a five-fold cross-validating of each model. TWAS associations (i.e., genes) were considered “transcriptome-wide significant” if they passed a strict Bonferroni-corrected threshold for all genes tested in the dataset (corrected significance *P* value: 0.05/3551 = 1.41 × 10^− 5^). Detailed description of the principle of FUSION and statistical model can be found in the original paper [56].

### Expression quantitative trait locus (eQTL) analysis

We performed eQTL analysis using 4 public available brain eQTL resources, including the CommonMind Consortium (CMC) [55], LIBD BrainSeq Phase II RNA-seq Project (LIBD2) [57], xQTL [58], and GTEx [50] project. Please refer to the original papers [50, 55, 57, 58] about the sample collection, RNA-seq data processing, and eQTL calculation.

### Differential expression analysis in schizophrenia cases and controls

We examined the expression level of genome-wide significant genes in brains of SCZ cases (*n* = 559) and controls (*n* = 936) by using the PsychEncode data [59]. Briefly, brain gene expression data of 559 schizophrenia cases and 936 controls were quantified and analyzed by the PsychEncode. Detailed information on tissue collection, RNA sequencing, gene expression quantification, and differential expression analysis were provided in PsychEncode papers [59] and website (http: http://resource.psychencode.org/).

## Results

### GWAS of Han Chinese cohort identified 2 new schizophrenia risk loci

**Fig. 1:**
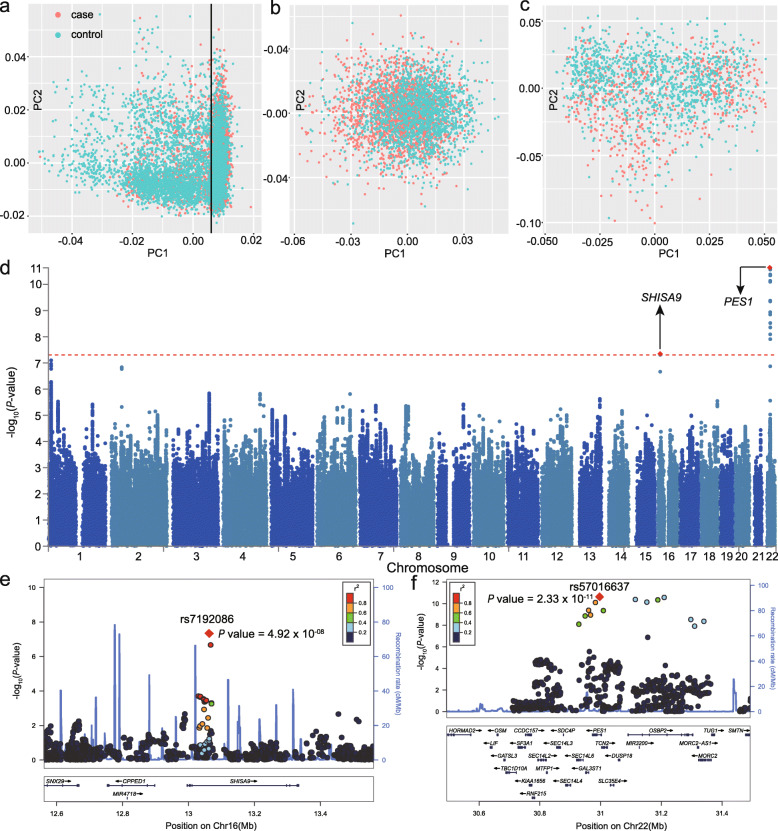
The genome-wide associations of our Han Chinese samples. **a** The principal component analysis (PCA) of cases and controls genotyped with ASA SNP array. **b** The PCA result of the ASA subgroup 1 (2,055 cases and 1,823 controls). **c** The PCA results of the ASA subgroup 2 (607 cases and 1,186 controls). **d** The Manhattan plot of meta-analysis results of our Han Chinese samples (3,493 cases and 4,709 controls). **e**,**f** The locuszoom plots of the two newly identified risk loci (the lead SNPs were rs7192086 and rs57016637)

Our samples had no overlap with the previously published SCZ GWAS of Han Chinese population [17, 19, 23, 60]. We first performed a PCA analysis using the samples genotyped with Illumina Asian Screening Arrays (ASA) and found population stratification of our samples (Fig. 1a); we thus divided our samples into two genetically matched subgroups. After stringent QC and excluding outliers, 2055 cases and 1823 controls were included in subgroup 1, and 607 cases and 1186 controls were included in subgroup 2 (Fig. 1b, c). For samples genotyped with GSA arrays, no obvious population stratification was observed. After strict QC, the final samples genotyped with GSA arrays included in this study were 831 cases and 1700 controls (Additional file 1: Figure S2). PCA analysis using genotype data of our samples and the 1000 Genomes project [38] showed that all of our samples clustered with samples of Han Chinese ancestry (Additional file 1: Figures S3, S4). After strict QC, imputation using the 1000 Genomes project phase 3 panel [45] and post-imputation QC, a total of 3,937,527 biallelic SNPs from 3493 SCZ cases and 4709 healthy controls were retained for GWAS. Principal components (PCs) were included as covariates [40, 41] (10 PCs for ASA subgroup1, 4 PCs for ASA subgroup2, 12 PCs for GSA samples) when performing GWAS. We firstly meta-analyzed the samples genotyped with ASA arrays (subgroups 1 and 2). We then conducted a meta-analysis through combining the results from ASA and GSA arrays with the fixed effect model. The genomic inflation (λ_GC_) of our combined meta-analysis (including ASA and GSA samples) was 1.10, and the λ_1000_ (scaled to a sample size of 1000 cases and 1000 controls) was 1.02 (which was very close to the reported values in previous Chinese GWAS [17, 23], 1.02 in Li et al. [23] and 1.019 in Yu et al. [17]) (Additional file 1: Figure S5), indicating that population stratification unlikely confounds our genome-wide association results.

Two genome-wide significant risk loci were identified in our sample (Fig. 1d). The lead risk SNP for the first locus is rs7192086 (*P* = 4.92 × 10^-08^, OR = 1.22), which is located in the intron 2 of the *SHISA9* gene (Fig. 1e). Of note, a previous study (7308 cases and 12,834 controls) showed nominal association between rs7192086 and SCZ (*P* = 1.34 × 10^− 05^, OR = 1.12) in European population [22]. The lead SNP for the second risk locus is rs57016637 (*P* = 2.33 × 10^−11^, OR = 1.34) located in the intron 2 of *PES1* (Fig. 1f). This genomic loci contain three independent significant SNPs, and the other two SNPs are rs117961127 (*P* = 2.73 × 10^−11^, OR = 1.35) (located in the intron 2 of *OSBP2*) and rs116976860 (1.42 × 10^−09^, OR = 1.33) (located in the intergenic region of *SEC14L6* and *GAL3ST1*). Of note, rs57016637 is an East Asian-specific polymorphism (Additional file 1: Figure S6).

### Meta-analysis with EAS and EUR identified 15 new risk loci

We then carried out a meta-analysis through meta-analyzing GWAS data obtained from our sample and a recent GWAS conducted in EAS [24]. Our meta-analysis using combined EAS samples (26,271 cases, 40,071 controls) [24] identified 24 risk loci (Additional file 1: Figure S7). However, all of these loci have been reported in a previous study [24]. Intriguingly, we noticed that rs3845188 (*P* = 6.50 × 10^−08^, OR = 0.91) (which is located in the intron 1 of the *NEBL* gene) reached the suggestive significance level (*P* = 5.00 × 10^−06^), suggesting this locus may be associated with SCZ (Additional file 1: Figure S8).

**Fig. 2:**
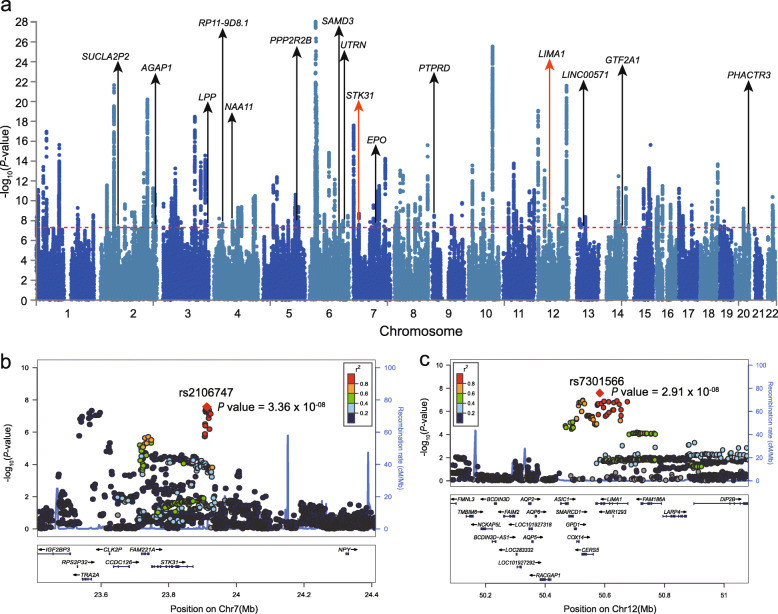
The genome-wide significant risk loci identified in the combined meta-analysis (a total of 59,911 cases and 83,527 controls). **a** The Manhattan plot of combined meta-analysis. **b** The locuszoom plot of the newly identified risk locus on 7p15.3 (the lead SNP is rs2106747). **c** The locuszoom plot of the newly identified risk locus on 12q13.12 (the lead SNP is rs7301566)

We further performed a meta-analysis through combining results of our study and GWAS results from EAS and PGC2 European samples (59,911 cases and 83,527 controls) [24]. We identified 153 genome-wide independent risk loci in the combined samples (Fig. 2a) (The definition of independent risk loci was described in methods section). Among these 153 risk loci, 15 were novel (Table 1, Additional file 1: Table S1). In total, we identified 17 new risk loci for SCZ.

**Table 1:** New genome-wide significant loci identified in this study (a total of 17 novel risk loci)

Genomic locus^a^	Lead SNP	Chr	Pos	A1/A2(MA^**b**^)	MAF_A^c^	MAF_U^d^	P	OR^e^	Nearby gene(s)^f^
**1**	**rs7192086**	**16**	**13061611**	**T/A(T)**	**0.32**	**0.28**	**4.92 × 10**^**−08**^	**1.22**	***SHISA9***
**2**	**rs57016637**	**22**	**30992925**	**G/C(G)**	**0.20**	**0.16**	**2.33 × 10**^**−11**^	**1.34**	***SF3A1, CCDC157, RNF215, SEC14L2, MTFP1, SEC14L3, SEC14L6, GAL3ST1, PES1, TCN2, SLC35E4, DUSP18, OSBP2, MORC2***
3	rs115487049	2	76297343	A/T(A)	0.042	0.036	4.72 × 10^**−**08^	1.07	*SUCLA2P2*
4	rs10178509	2	236792838	T/C(T)	0.39	0.42	1.08 × 10^**−**08^	0.95	*AGAP1*
5	rs1426271	3	188187250	A/G(G)	0.35	0.33	3.23 × 10^**−**08^	0.95	*LPP*
6	rs2911914	4	37820679	T/G(T)	0.25	0.27	2.12 × 10^**−**08^	0.95	*PGM2*
7	rs6848123	4	80203425	A/C(C)	0.14	0.16	1.17 × 10^**−**08^	1.05	*NAA11*
8	rs319227	5	146245762	A/C(A)	0.33	0.30	6.11 × 10^**−**09^	1.05	*PPP2R2B*
9	rs12202107	6	130598623	T/C(C)	0.42	0.45	2.51 × 10^**−**08^	1.05	*SAMD3,TMEM200A*
10	rs9386072	6	144859509	A/T(A)	0.43	0.47	1.01 × 10^**−**08^	0.93	*UTRN*
11	rs2106747	7	23911499	A/G(G)	0.40	0.42	3.36 × 10^**−**08^	1.05	*TRA2A, FAM221A, STK31*
12	rs492430	7	100313099	T/G(T)	0.065	0.058	2.26 × 10^**−**08^	1.07	*ACTL6B, GNB2, GIGYF1, POP7, EPO*
13	rs59761926	9	10238178	T/C(T)	0.37	0.39	3.02 × 10^**−**09^	0.95	*PTPRD*
14	rs7301566	12	50581647	T/C(T)	0.099	0.091	2.91 × 10^**−**08^	1.06	*ASIC1, SMARCD1, GPD1, COX14, CERS5, LIMA1*
15	rs6563592	13	38814939	T/G(G)	0.31	0.30	2.13 × 10^**−**08^	0.95	*LINC00571*
16	rs2099108	14	81675420	C/G(G)	0.45	0.47	2.67 × 10^**−**08^	1.05	*GTF2A1,STON2*
17	rs6100546	20	58252407	T/C(C)	0.079	0.076	2.79 × 10^**−**08^	0.94	*PHACTR3*

^a^ The new loci identified in our Han Chinese cohort are shown in bold. ^b^ minor allele of our Chinese samples. ^c^ minor allele frequency in our Chinese cases. ^d^ minor allele frequency in our Chinese controls. ^e^ Odds ratio is based on A1; ^f^ The nearby genes were defined with FUMA. FUMA firstly identified the SNPs that were in LD (r^2^ > 0.6) with independent lead SNPs. And these SNPs were further used to identify the nearby genes by FUMA with default parameters (posMapWindowSize = 10 kb). More details about defining nearby genes can be found in FUMA website: https://fuma.ctglab.nl/tutorial#parameters

### Identification of potential target genes of the newly identified risk SNPs

To identify the potential target genes of the newly identified risk SNPs, we performed eQTL analysis using data from the human brain tissues. Among the 17 lead SNPs, 9 showed associations (uncorrected *P* < 0.05) with expression of 33 genes in the human brain (Additional file 1: Table S2). Of note, rs2106747 (*P* = 3.36 × 10^−08^, OR = 1.05, Fig. 2b) showed a strong association with *FAM221A* expression in all four eQTL datasets. Another interesting SNP is rs7301566 (*P* = 2.91 × 10^−08^, OR = 1.06, Fig. 2c), which was associated with expression of several genes, including *COX14, DIP2B, CERS5, RP4-605O3.4, SPATS2, ASIC1,* and *ATF1* (Additional file 1: Table S2). These results suggest that the newly identified risk variants may contribute to SCZ risk through regulating expression of these eQTL genes.

### Differential expression analysis in schizophrenia cases and controls

We explored the expression level of potential target genes of the newly identified risk variants in SCZ cases and controls using expression data from the PsychEncode (including 559 cases and 936 controls) [59]. Among the 33 potential target genes, 14 showed nominal difference in expression in SCZ cases compared with controls (Additional file 1: Table S3), supporting that the newly identified risk variants may confer SCZ risk by regulating the expression of these target genes. In addition, we also examined the expression of genes located near the two newly identified loci in our Chinese samples (Table 1, Fig. 1e, f). We found that *SHISA9* showed a trend of upregulation in brains of SCZ cases compared with controls (*P* = 0.053). Interestingly, *OSBP2* was significantly downregulated in brains of SCZ cases compared to controls (*P* = 1.07 × 10^−07^). Taken together, these expression data provide further evidence that support the newly identified risk variants may confer risk of SCZ through modulating expression level of these genes.

### Polygenic risk score (PRS) profiling

**Fig. 3:**
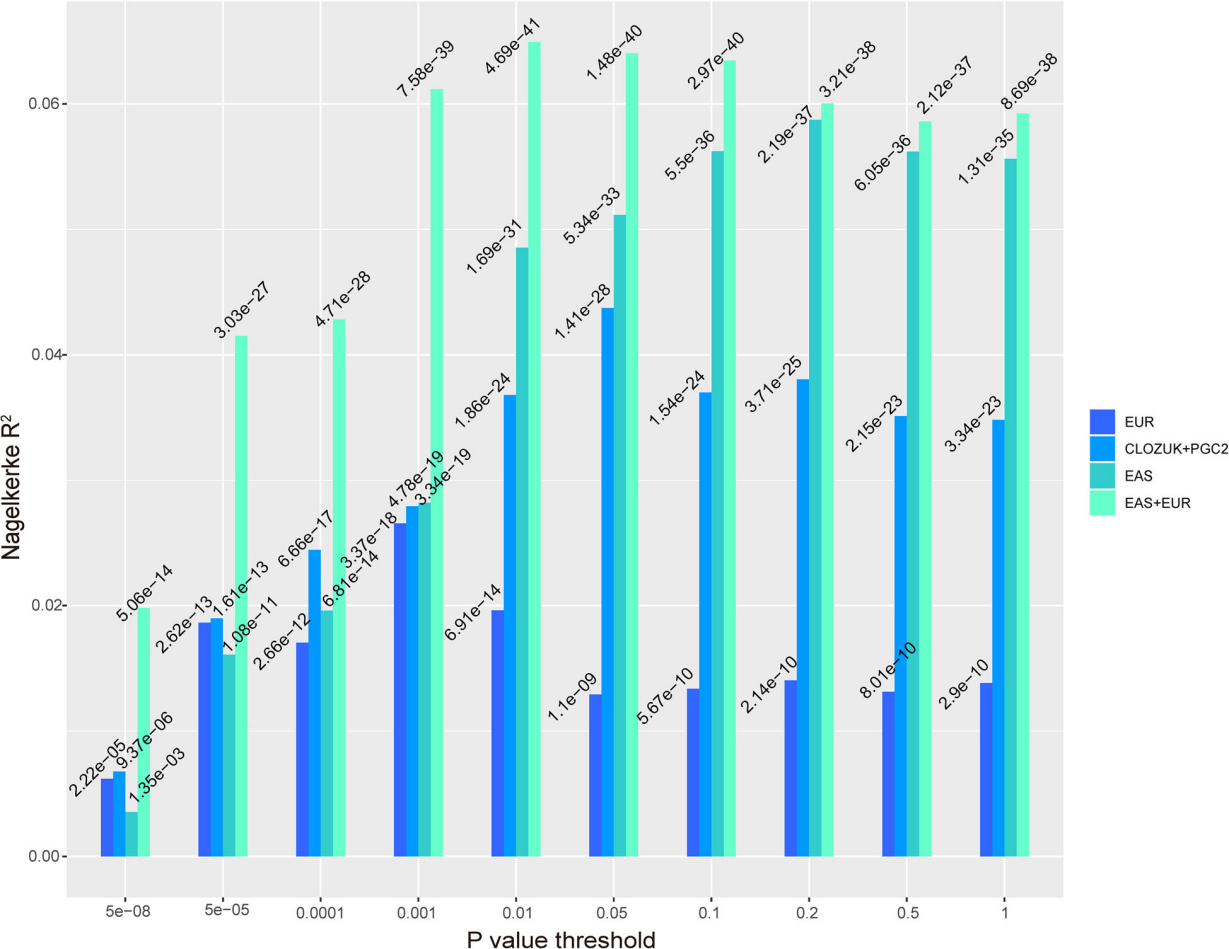
PRS analysis results of our ASA subgroup 1 samples. PRSs were computed using GWAS summary statistics from four training sets. The first is EAS (EAS) training set (including 22,778 SCZ cases and 35,362 controls). The second is EAS + PGC2 European (EAS + EUR) training set (including 56,418 SCZ cases and 78,818 controls). The third is PGC2 European (EUR) training set (33,640 cases and 43,456 controls), and the fourth is CLOZUK+PGC2 (CLOZUK+PGC2) training set (40,675 SCZ cases and 64,643 controls). The trans-ancestry EAS + EUR data set had better performance at different *P* thresholds

We conducted PRS analysis to predict the case-control status of our samples (ASA subgroup 1) and estimate the phenotypic variance (of our samples) that can be explained by the published GWAS summary statistics data [24]. When using the summary statistics from EAS as training set [24], the explained variance (estimated by Nagelkerke R^2^) ranged from 0.4 to 5.9% and the training data has the largest variance explanation at *P* value = 0.2 (*P* = 2.19 × 10^−37^) (Fig. 3). The training set from EAS + EUR has an overall better prediction performance and variance explanation at each *P* value threshold than EAS [24], and the explained variance ranged from 2.0% to 6.5%. At *P* value = 0.01 threshold, the training dataset has the largest variance explanation (*P* = 4.69 × 10^−41^). The EUR and CLOZUK+PGC2 training sets had relatively poor prediction performance than the EAS and EAS + EUR training set. The explained variance (estimated by Nagelkerke R^2^) ranged from 0.6 to 2.7% and 0.7 to 4.4% for EUR and CLOZUK+PGC2 training datasets, respectively. The PRS analysis indicated that EAS and EAS + EUR training sets had relative good power to predict the SCZ and healthy controls status of our sample, and the EAS + EUR training set had better prediction performance.

### Identification of tissues and cell types associated with schizophrenia

We conducted tissue enrichment analysis by using GWAS associations from the combined samples (i.e., our cohort, EAS and PGC2 EUR [24]) and gene expression from GTEx [50], with the use of FUMA [46]. SCZ heritability was mainly enriched in brain tissues (Additional file 1: Figure S9a). Of note, two brain cerebellum tissues showed the strongest associations (brain cerebellar hemisphere, *P* = 2.54 × 10^−23^, and brain cerebellum, *P* = 8.51 × 10^−23^). The frontal cortex (BA9) (*P* = 1.28 × 10^−19^) and brain cortex (*P* = 2.20 × 10^−18^) also showed significant enrichment.

We further conducted cell-type enrichment analysis (Additional file 1: Figure S9c). Similar with previous findings [52], two telencephalon neuronal cell types, including telencephalon projecting excitatory interneurons (*P* = 1.12 × 10^−09^, FDR < 0.05) telencephalon projecting inhibitory interneurons (*P* = 1.15 × 10^−06^, FDR < 0.05), showed significant enrichment for SCZ associations. We also identified other novel associations, including oligodendrocytes (*P* = 8.03 × 10^−07^, FDR < 0.05), cholinergic and monoaminergic neurons (*P* = 6.06 × 10^−05^, FDR < 0.05), and cerebellum neurons (*P* = 4.99 × 10^−05^, FDR < 0.05). Collectively, our results suggest that SCZ risk genes are actively expressed in these identified tissues and cell types, implying the pivotal roles of these tissues and cell types in SCZ.

### Gene set analysis identified enriched gene sets and pathways

We carried out gene set enrichment analysis and identified 6 significant enriched terms, including neuron spine (*P* = 7.06 × 10^−07^, FDR = 0.0044), membrane depolarization during action potential (*P* = 4.72 × 10^−06^, FDR = 0.015), cytosolic calcium ion transport (*P* = 4.03 × 10^−05^, FDR = 0.045), voltage gated sodium channel complex (*P* = 4.09 × 10^−05^, FDR = 0.045), t tubule (*P* = 4.32 × 10^−05^, FDR = 0.045), and voltage gated sodium channel activity (*P* = 2.57 × 10^−05^, FDR = 0.045). In addition, a few gene sets also showed a trend of enrichment, including regulation of synaptic plasticity (*P* = 1.5 × 10^−04^, FDR = 0.074) and neurotransmitter receptor complex (*P* = 2.00 × 10^−04^, FDR = 0.074) (Additional file 1: Table S4).

### Enrichment of schizophrenia heritability in conserved genomic regions, promoters, and enhancers

LDSC analysis showed that SCZ associations were mainly enriched in various conserved genomic regions, promoters, and enhancers (SuperEnhancer_Hnisz, H3K27ac_Hnisz) (Additional file 1: Figure S9b), which were consistent with previous findings [26, 54].

### TWAS analysis identified risk genes for schizophrenia

**Fig. 4:**
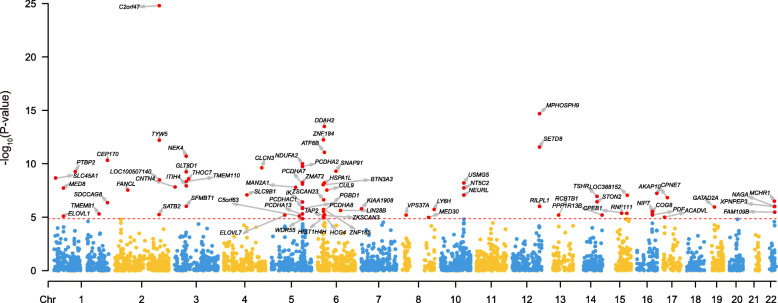
TWAS results. The red line indicates the significant level corrected by the Bonferroni test. The summary associations from all combined samples (including this study, EAS and EUR) were used to perform TWAS

We conducted a TWAS to identify genes whose cis-regulated expression were associated with SCZ. We identified 76 transcriptome-wide significant genes (corrected by multiple comparison testing) (Fig. 4 and Additional file 1: Table S5). Among these identified genes, 24 have been reported in a previous study [59]. Of note, *GIGYF1* (*P* = 3.03 × 10^−05^) (located near the newly identified risk loci) showed a trend of TWAS significance (Additional file 1: Table S5). Taken together, our TWAS identified 52 new risk genes whose cis-regulated expression change may have a role in SCZ.

## Discussion

In this study, we first performed a GWAS for SCZ in Han Chinese samples. We then conducted meta-analyses by combining our results and the published GWAS summary statistics from individuals of East Asian and European ancestry [24]. We identified 2 new genome-wide significant risk loci in our Han Chinese cohort. SNP rs7192086 was located in the intron 2 of the *SHISA9* gene. SHISA9 (also known as CKAMP44) protein is a brain-specific type-I transmembrane protein and is highly expressed in hippocampal dentate gyrus and brain cerebral cortex [61]. SHISA9 is enriched at postsynaptic sites and its intracellular domain contains a PDZ domain interaction site which could physically interact with AMPA-type glutamate receptor (AMPAR); thus, SHISA9 plays important roles in synaptic short-term plasticity [61]. Notably, the AMPAR shows abnormal forward trafficking in the frontal cortex of SCZ patients [62]. These lines of evidence suggest that *SHISA9* may contribute to SCZ by affecting the function of AMPAR and synaptic transmission. However, further functional studies are warranted to reveal the role of *SHISA9* in SCZ.

Another genome-wide significant risk variant identified in our sample is rs57016637. Intriguingly, we noticed that rs57016637 is fixed in other populations (Figure S8). Despite the fact that the majority of risk variants have similar effects between EUR and EAS populations [23], population heterogeneity still exists. For example, rs374528934 was reported to be strongly associated with SCZ in EAS (*P* = 5 × 10^−11^). Nevertheless, the MAF of rs374528934 in EUR is quite low (0.7%) [24]. Our data suggest that rs57016637 may be a Han Chinese-specific risk variant for SCZ. SNP rs117961127 (in LD with lead SNP rs57016637 in the loci, r^2^ = 0.32 in 1000 East Asian samples) was located in the intron 2 of *OSBP2*, a gene that encodes a cholesterol-binding protein. Cholesterol levels were reported to be altered in SCZ cases compared to controls [63]. In addition, Krakowski et al. showed that cholesterol levels were strongly associated with cognition in SCZ [64]. These data suggest that *OSBP2* may have a role in SCZ through regulating cholesterol levels. Further investigating the role of *OSBP2* in SCZ is needed.

Two novel GWAS loci reported in our analysis did not reach GWS level in our follow-up meta-analysis with EAS and EAS + EUR samples (Additional file 1: Table S1). Of note, previous studies have also observed similar results in GWAS studies of Han Chinese [19, 60]. For example, the top associations identified by Shi et al. (rs16887244, rs10489202) [19] and Yue et al. (rs1233710, rs1635, rs2142731, rs11038167, rs11038172, rs835784) [60] in Chinese population did not reach genome-wide significance level in a larger meta-analysis (in EAS) reported by Lam et al*.* [24]. More work is needed to explore if this observation is due to population-specific associations or genetic heterogeneity between regional samples.

By meta-analyzing our results with GWAS associations from EAS and EUR [24], we identified 15 new risk loci, including 7p15.3 (the lead risk SNP is rs2106747, which was strongly associated with the expression level of *FAM211A*) and 12q13.12 (the lead risk SNP is rs7301566, which was an eQTL of several genes, including *COX14, DIP2B, CERS5, RP4-605O3.4, SPATS2, ASIC1,* and *ATF1*)*.* These new risk loci provide valuable clues for further functional study. Further functional investigation of these risk genes will also provide important insights into SCZ pathogenesis and help to develop potential therapeutic targets. Some of our newly identified GWS loci are located in genomic regions near previous reported loci. For example, rs319227 (*P* = 6.11 × 10^−09^, Table 1) and rs11958187 (*P* = 9.39 × 10^−09^, reported by Lam et al. [24]) are located near *PPP2R2B*, but these two index SNPs are not in LD (R^2^ < 0.1). In addition, rs2106747 (*P* = 3.36 × 10^−08^, Table 1) and rs112316332 (*P* = 3.04 × 10^−08^, reported by Lam et al. [24]) also showed similar results. These results suggest that these risk loci are genetically independent. However, more work is warranted to elucidate the functional mechanisms of these loci.

We compared our results with the findings reported by PGC3 (preprinted on medRxiv) [65]. We found that 6 loci (index SNPs are rs115487049, rs6848123, rs319227, rs59761926, rs7301566, rs6563592) reported in our study (Table 1) also show significant associations with SCZ in PGC3. This observation suggested though our sample size is relatively small, it could help us to discover new associations. A meta-analysis with PGC3 will help to identify more new associations and the underlying genetic basis of SCZ

Despite the fact that SCZ risk associations were highly shared between EAS and EUR, conducting GWAS in EAS is still important as it could improve our understanding of the underlying biology of SCZ. Firstly, Lam et al. showed that the genetic correlation between the EAS and EUR GWAS summary statistics is 0.98, indicating that the genetic basis of SCZ are highly shared between EAS and EUR. With the increasing EAS sample, novel risk loci well be identified continuously, which will help us to understand the genetic basis of SCZ better. In addition, Lam et al. also reported EAS-specific association, e.g., rs374528934 (*P* = 5 × 10^− 11^, minor allele frequency is 0.45 and 0.007 in EAS and EUR, respectively). These results demonstrated that the EAS GWAS summary statistics can not only facilitate to discover the genetic associations shared between EAS and EUR, but also help to identify EAS-specific GWAS associations. Finally, genome-wide associations from different populations help to improve fine-mapping [24]. We believe that EAS GWAS summary statistics will provide important insights into the genetic architecture (both shared with EUR population and EAS-specific) and the underlying biology of SCZ.

In the meta-analysis of EAS samples (our sample + EAS) [24], we found some novel loci (compared with EAS summary statistics alone). However, these loci also reached genome-wide significance level in EAS + PGC2 EUR summary statistics [24]. For example, rs12031518 reached genome-wide significance level in our EAS meta-analysis (our sample + EAS) (*P* = 4.96 × 10^−08^). Of note, this SNP did not reach genome-wide significance level in EAS samples (*P* = 7.58 × 10^−08^). However, it showed genome-wide significant association in EAS + PGC2 EUR summary statistics (*P* = 6.43 × 10^−11^) [24]. We calculated the genetic correlation between our ASA/GSA samples and the reported EAS (22,778 cases; 35,362 controls) [24]. Although these two GWAS summary statistics are highly correlated (the genetic correlation is 0.71), the genetic correlation is not very close to 1. A possible reason is that the sample size included in our study is relatively small (3493 cases and 4709 controls) compared with the reported EAS samples (22,778 case and 35,362 controls).

PRS analysis revealed several interesting results. First, EAS training set had overall better performance than EUR and CLOZUK+PGC2 samples (though the sample size of EAS is less than the two GWAS summary statistics), indicating that similar ethnic background (of the EAS summary statistics) helps to improve the PRS prediction performance. Second, CLOZUK+PGC2 training set had better performance than EUR, indicating that the training set with larger sample size had better performance. Third, EAS + EUR GWAS summary statistics had the best performance than other training sets. This result reflects that trans-ancestry meta-analysis improves the prediction power.

Tissue and cell-type enrichment analysis revealed that SCZ associations showed the significant enrichment in the cerebellum, suggesting the potential role of cerebellum in SCZ. Of note, several previous studies also suggested that cerebellum may play an important role in SCZ [66–69]. These results suggest that the cerebellum may have a pivotal role in SCZ etiology.

Our study has several limitations. Firstly, our sample size is relatively small compared with recent SCZ GWAS cohort, such as PGC2 [26], Clozuk [21], or East Asian meta-analysis [24]. Additional SCZ risk loci will be found with the increase of sample size. Secondly, although we reported 17 novel risk loci, the casual variants and genes of these identified risk loci remain largely unknown. Further work, including pinpointing causal variants and genes, functional characterization of risk genes, exploring the role of risk genes in developing and adult brain, will provide pivotal insights into SCZ pathophysiology. Thirdly, we used eQTL data from Europeans to explore the associations between genome-wide significant SNPs and gene expression level in human brain. Considering that some novel risk loci were from our Chinese cohort, an ideal approach is to check the effect of the novel genetic variations and gene expression both in EAS and EUR populations. However, the brain eQTL data in EAS is not publically available so far. More work is needed to explore if these genetic variations also associated with gene expression in Chinese population. Fourthly, although including PCs as covariate is a regular and useful way to correct population stratification of GWAS, challenges remain in PCA. For example, selecting the optimal number of PCs [70] remains an open question (i.e., is relatively arbitrary, different numbers of PCs were reported in different studies). In addition, more work is needed to determine the number of optimal genetic markers for PC calculation.

## Conclusions

In summary, we identified 17 risk loci for SCZ, including a Han Chinese-specific risk locus. We carried out comprehensive post-GWAS analysis, including TWAS, eQTL, differential expression analysis, and heritability partitioning (LDSC). Our results expand the list of genome-wide significant risk loci for SCZ and provide new insight into genetic architecture of SCZ.

## Supplementary Information

**Additional file 1: Figure S1.** The flowchart of our quality control steps. **Figure S2.** The PCA results of the GSA group (831 cases and 1,700 controls). **Figure S3.** The PCA results of our samples (genotyped with ASA SNP array) and subjects from the 1000 Genome project (including CHB, CHS, JPT, CEU and YRI). **Figure S4.** The PCA results of our samples (genotyped with GSA SNP array) and subjects from the 1000 Genome project (including CHB, CHS, JPT, CEU and YRI). **Figure S5.** The Quantile-Quantile plots of our Han Chinese samples. **Figure S6.** The allelic frequency of rs57016637 in global populations from the 1000 Genome project. **Figure S7.** The Manhattan plot of meta-analysis result of our Han Chinese samples and East Asian samples (26,271 cases and 40,071 controls). **Figure S8.** The locuszoom plot of rs3845188 (*P* = 6.50 × 10^-8^, OR = 0.91). **Figure S9.** Tissue and cell-type enrichment results. **Table S1.** The detail association result of the new genome wide significant loci identified in this study in different meta-analysis datasets. **Table S2.** Genes associated with the 17 newly identified lead SNPs in the human brain tissues. **Table S3.** Expression analysis of the potential eQTL target genes (of the newly identified lead SNPs) in schizophrenia cases and controls. **Table S4.** The MAGMA gene set enrichment analysis result (items with FDR < 0.10 were listed). **Table S5.** The TWAS result. Significant genes (after Bonferroni correction) were listed.

## Data Availability

The datasets generated and/or analyzed during the current study are not publicly available due to the regulations of the Ministry of Science and Technology of the People’s Republic of China (on human genetic resource usage). We need to comply with relevant regulations regarding the release of original genetic data (including the summary statistics) of the Chinese patients. This will take some time to finish according to the related policy. Data depositing in a third-party website (without application) is not allowed before approval. We understand that sharing of the genome-wide summary statistics publicly will contribute to the progress of the relevant field and we are planning to promote the progress of sharing summary statistics after this study is finalized. In addition, these GWAS samples were recruited from multiple hospitals across Mainland China, and this study could not have been completed without the great efforts from all the collaborators, we will need to get approval from all the authors (and their affiliated agencies) before sharing the summary statistics. However, these data are available from the corresponding author on reasonable request. URLs: PLINK, http://www.cog-genomics.org/plink2; KING, http://people.virginia.edu/~wc9c/KING/; EIGENSOFT, https://www.hsph.harvard.edu/alkes-price/software/; GCTA, http://cnsgenomics.com/software/gcta/; The 1000 Genome project website, https://www.internationalgenome.org/; Minimac3, https://genome.sph.umich.edu/wiki/Minimac3; LDSC, https://github.com/bulik/ldsc; MAGMA, https://ctg.cncr.nl/software/magma; MSigDB, http://software.broadinstitute.org/gsea/msigdb/; PsychEncode, http://www.psychencode.org/;; Locuszoom, http://locuszoom.org; FUMA, https://fuma.ctglab.nl/. Single cell RNA-seq analysis, https://github.com/jbryois/scRNA_disease; PRSice2, http://www.prsice.info/; FUSION, http://gusevlab.org/projects/fusion/; SZDB, http://www.szdb.org

## Abbreviations

ASA: Asian Screening Array
CMC: CommonMind Consortium
EAS: East Asian ancestry
eQTL: Expression quantitative trait locus
EUR: European ancestry
GSA: Global Screening Array
GWAS: Genome-wide association studies
LDSC: Linkage disequilibrium score regression
PCA: Principal component analysis
PRS: Polygenic risk score
SCZ: Schizophrenia
TWAS: Transcriptome-wide association study
