# Supplementary material for: Genome-wide association study followed by trans-ancestry meta-analysis identify 17 new risk loci for schizophrenia
Source: BMC Med. 2021 Aug 12;19:177. doi: 10.1186/s12916-021-02039-9 (PMC8359304; doi:10.1186/s12916-021-02039-9)
Supplement: Supplementary file 1 — Additional file 1: Figure S1. The flowchart of our quality control steps. Figure S2. The PCA results of the GSA group (831 cases and 1,700 controls). Figure S3. The PCA results of our samples (genotyped with ASA SNP array) and subjects from the 1000 Genome project (including CHB, CHS, JPT, CEU and YRI). Figure S4. The PCA results of our samples (genotyped with GSA SNP array) and subjects from the 1000 Genome project (including CHB, CHS, JPT, CEU and YRI). Figure S5. The Quantile-Quantile plots of our Han Chinese samples. Figure S6. The allelic frequency of rs57016637 in global populations from the 1000 Genome project. Figure S7. The Manhattan plot of meta-analysis result of our Han Chinese samples and East Asian samples (26,271 cases and 40,071 controls). Figure S8. The locuszoom plot of rs3845188 (P = 6.50 × 10^-8^, OR = 0.91). Figure S9. Tissue and cell-type enrichment results. Table S1. The detail association result of the new genome wide significant loci identified in this study in different meta-analysis datasets. Table S2. Genes associated with the 17 newly identified lead SNPs in the human brain tissues. Table S3. Expression analysis of the potential eQTL target genes (of the newly identified lead SNPs) in schizophrenia cases and controls. Table S4. The MAGMA gene set enrichment analysis result (items with FDR < 0.10 were listed). Table S5. The TWAS result. Significant genes (after Bonferroni correction) were listed. [file 12916_2021_2039_MOESM1_ESM.doc]

**Supplementary Material for**

**Genome-wide association study followed by trans-ancestry meta-analysis identify 17 new risk loci for schizophrenia**

Jiewei Liu, Shiwu Li, Xiaoyan Li, Wenqiang Li, Yongfeng Yang, Suqin Guo, Luxian Lv, Xiao Xiao, Yong-Gang Yao, Fanglin Guan, Ming Li, & Xiong-Jian Luo^*^

**Supplementary tables and figures**

**Figure S1:** The flowchart of our quality control steps.

**Figure S2:** The PCA results of the GSA group (831 cases and 1,700 controls).

**Figure S3:** The PCA results of our samples (genotyped with ASA SNP array) and subjects from the 1000 Genome project (including CHB, CHS, JPT, CEU and YRI).

**Figure S4:** The PCA results of our samples (genotyped with GSA SNP array) and subjects from the 1000 Genome project (including CHB, CHS, JPT, CEU and YRI)

**Figure S5:** The Quantile-Quantile plots of our Han Chinese samples

**Figure S6:** The allelic frequency of rs57016637 in global populations from the 1000 Genome project

**Figure S7:** The manhattan plot of meta-analysis result of our Han Chinese samples and East Asian samples (26,271 cases and 40,071 controls)

**Figure S8:** The locuszoom plot of rs3845188 (P=6.50×10^-8^, OR=0.91)

**Figure S9:** Tissue and cell-type enrichment results

**Table S1:** The detail association result of the new genome wide significant loci identified in this study in different meta-analysis datasets

**Table S2:** Genes associated with the 17 newly identified lead SNPs in the human brain tissues

**Table S3:** Expression analysis of the potential eQTL target genes (of the newly identified lead SNPs) in schizophrenia cases and controls

**Table S4:** The MAGMA gene set enrichment analysis result (items with FDR<0.10 were listed).

**Table S5:** The TWAS result. Significant genes (after Bofferoni correction) were listed


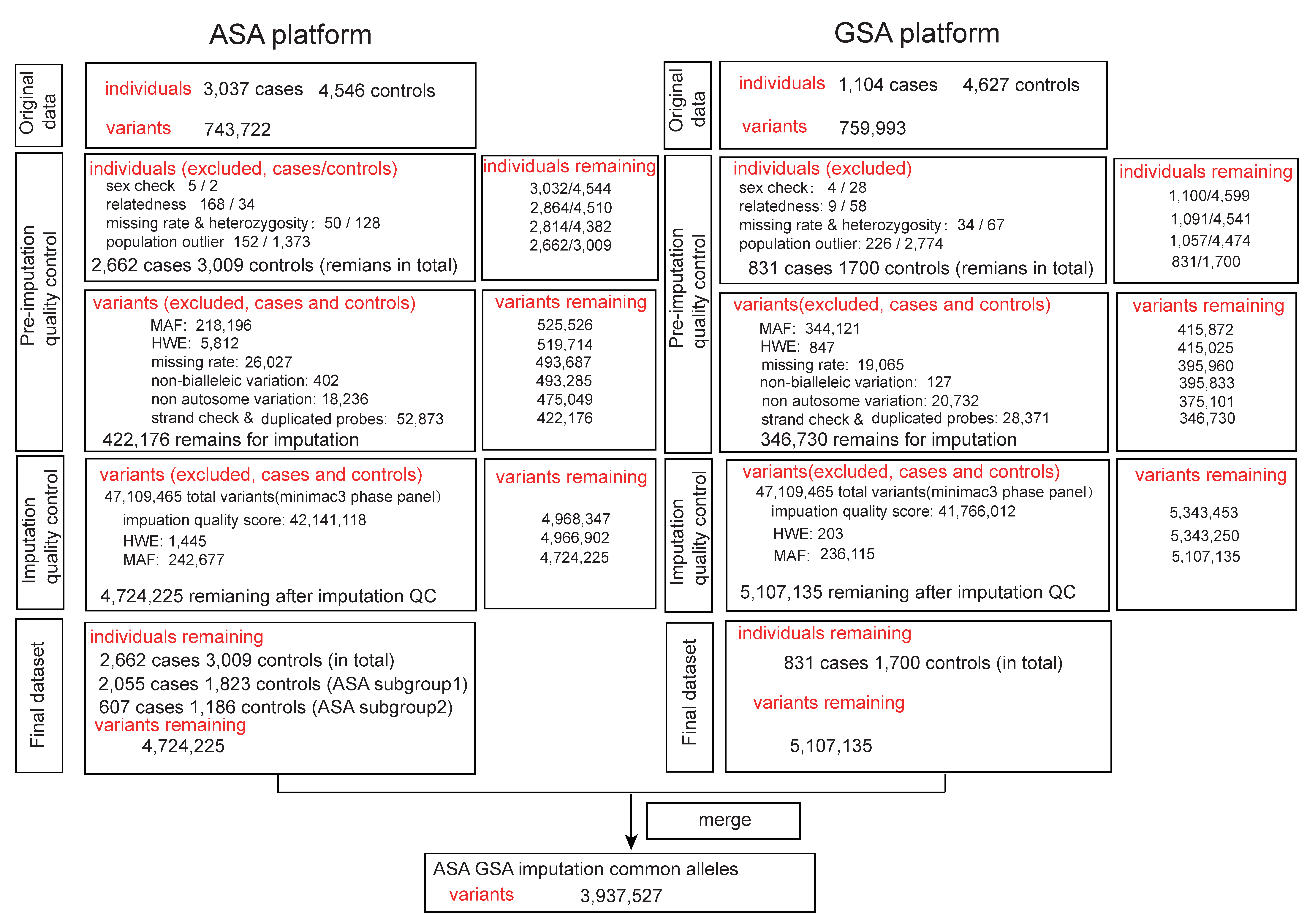


**Figure S1. The flowchart of our quality control steps.**


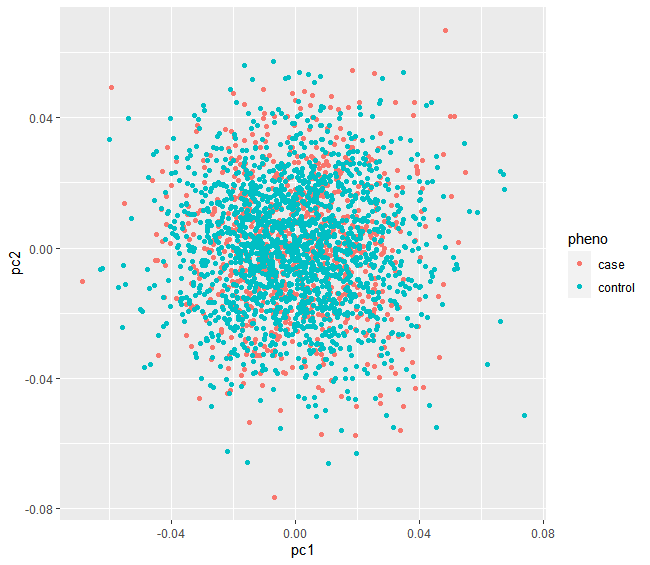


**Figure S2. The PCA results of the GSA group (831 cases and 1,700 controls).**


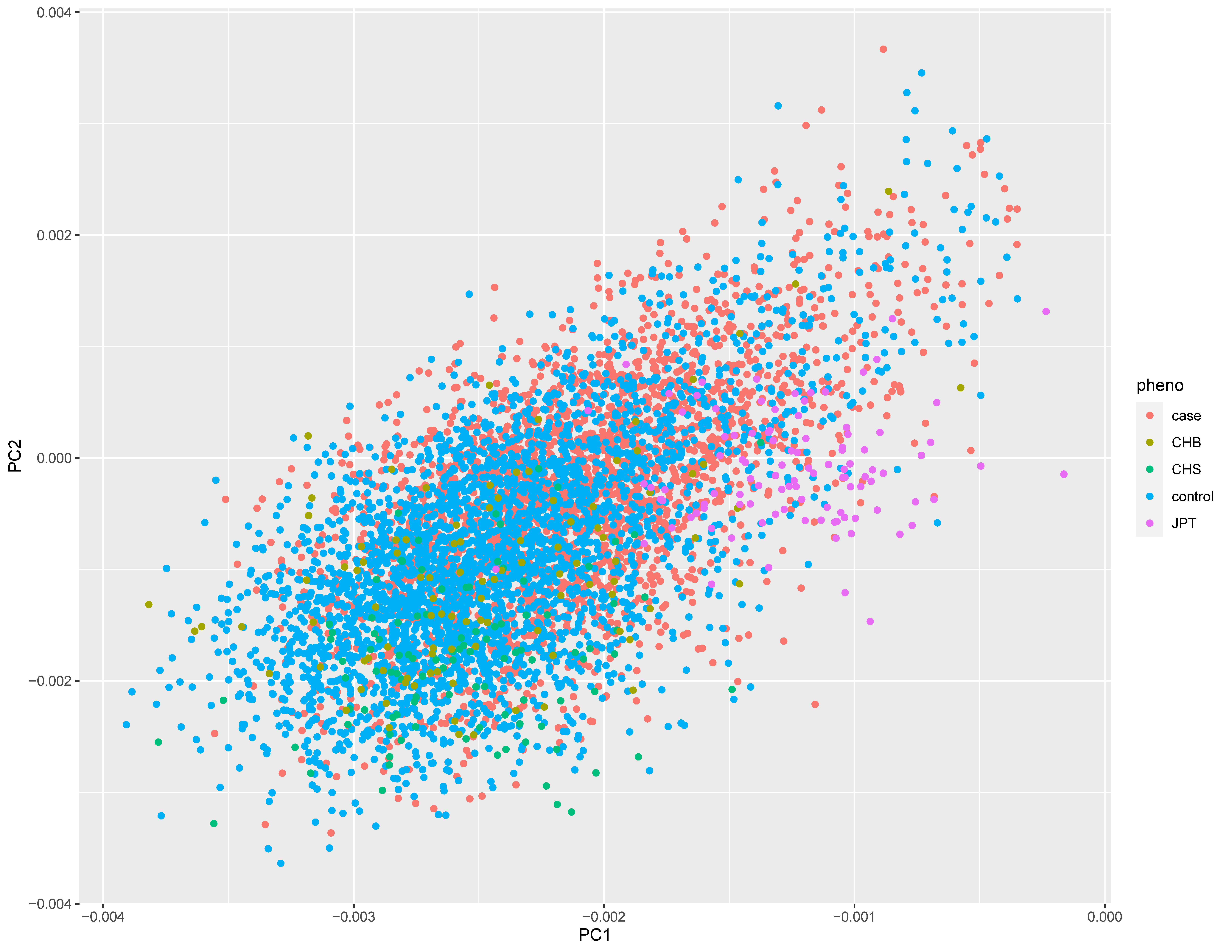

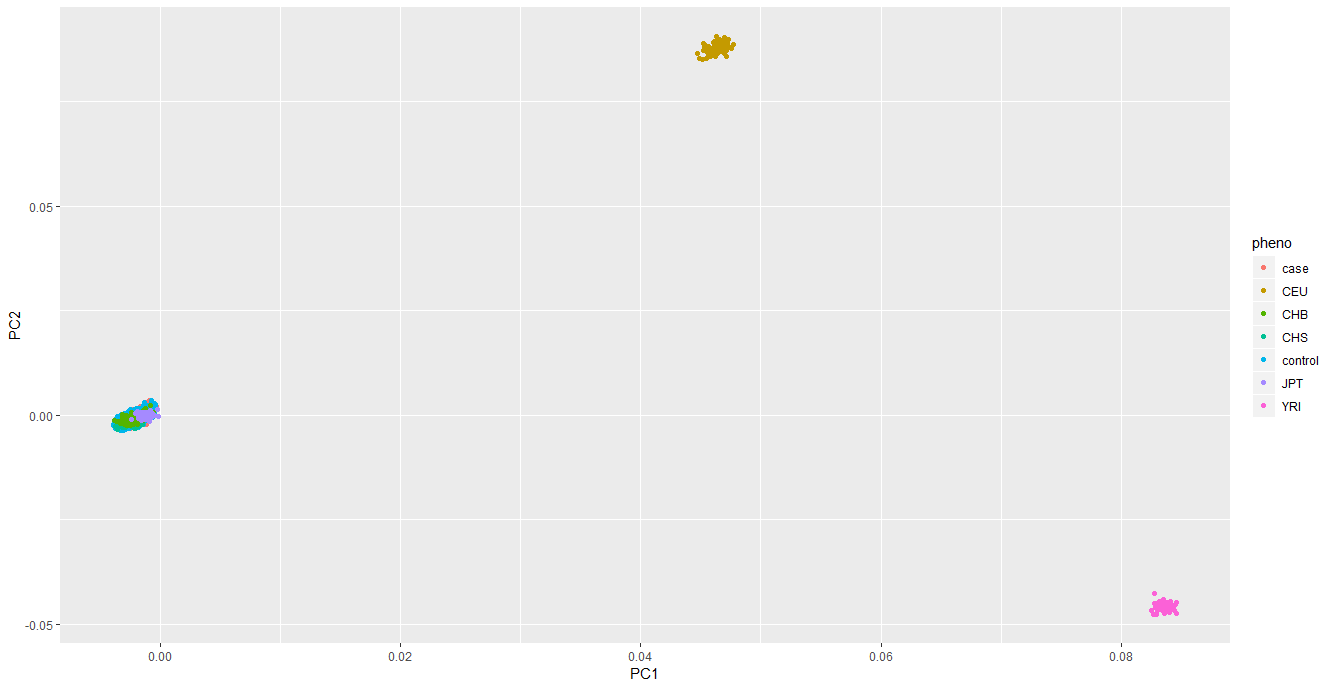


**Figure S3. The PCA results of our samples (genotyped with ASA SNP array) and subjects from the 1000 Genome project (including CHB, CHS, JPT, CEU and YRI).**


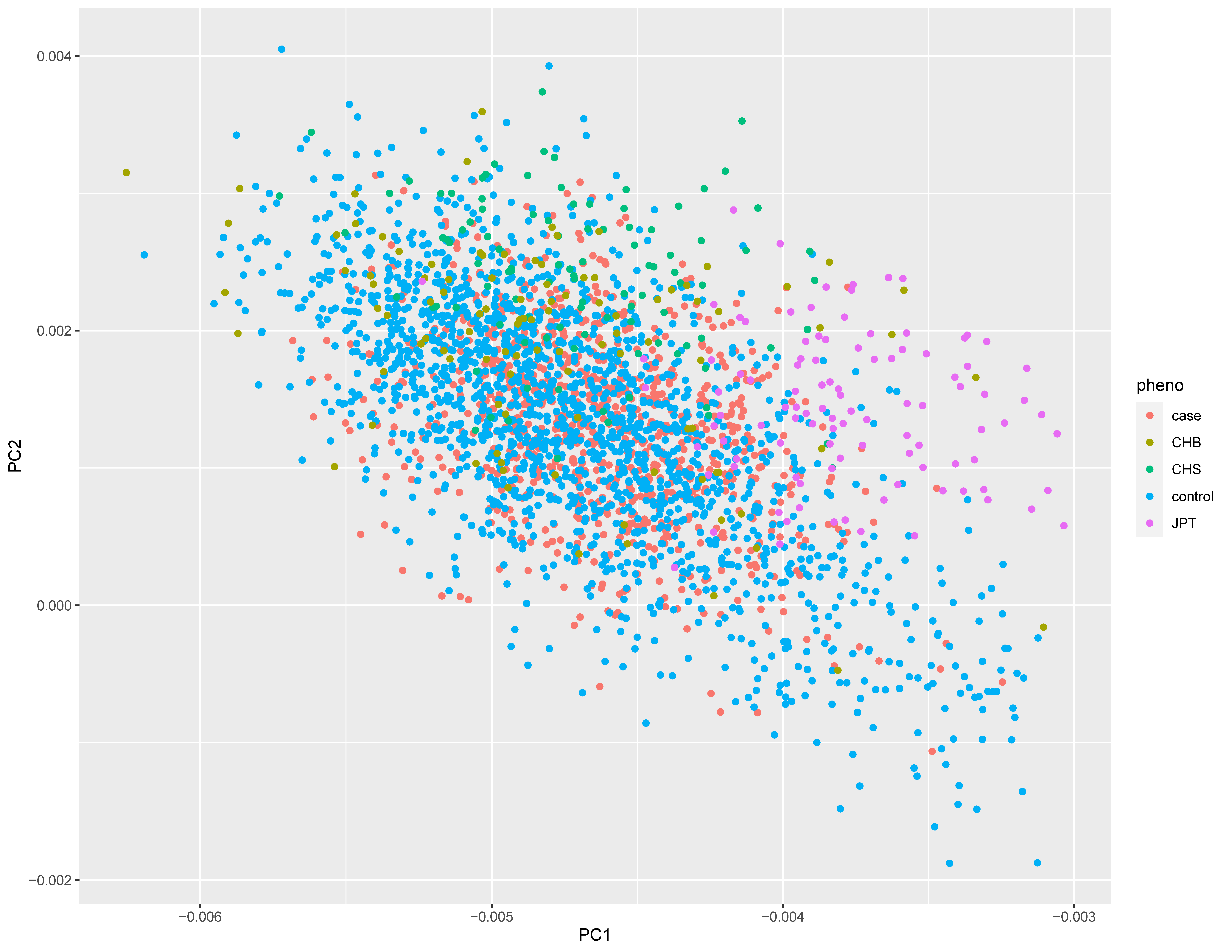

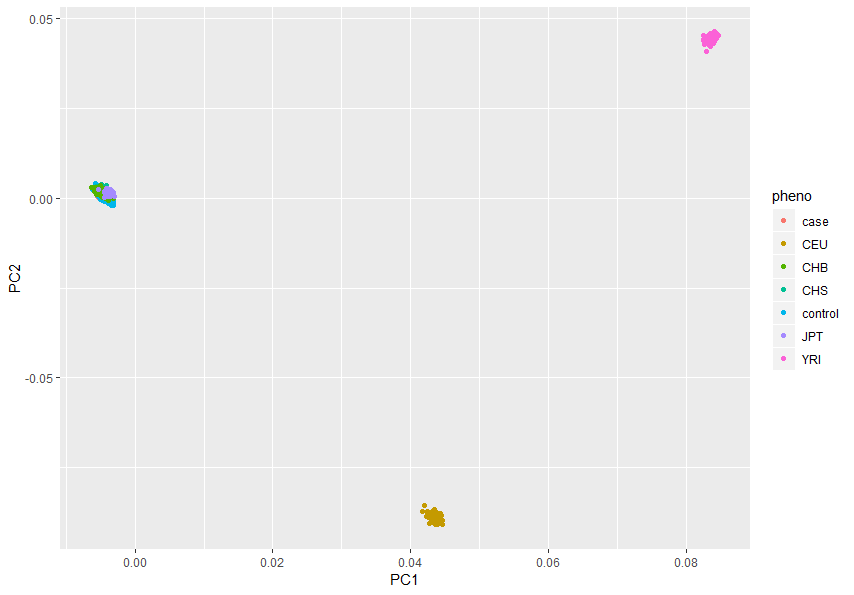


**Figure S4. The PCA results of our samples (genotyped with GSA SNP array) and subjects from the 1000 Genome project (including CHB, CHS, JPT, CEU and YRI).**


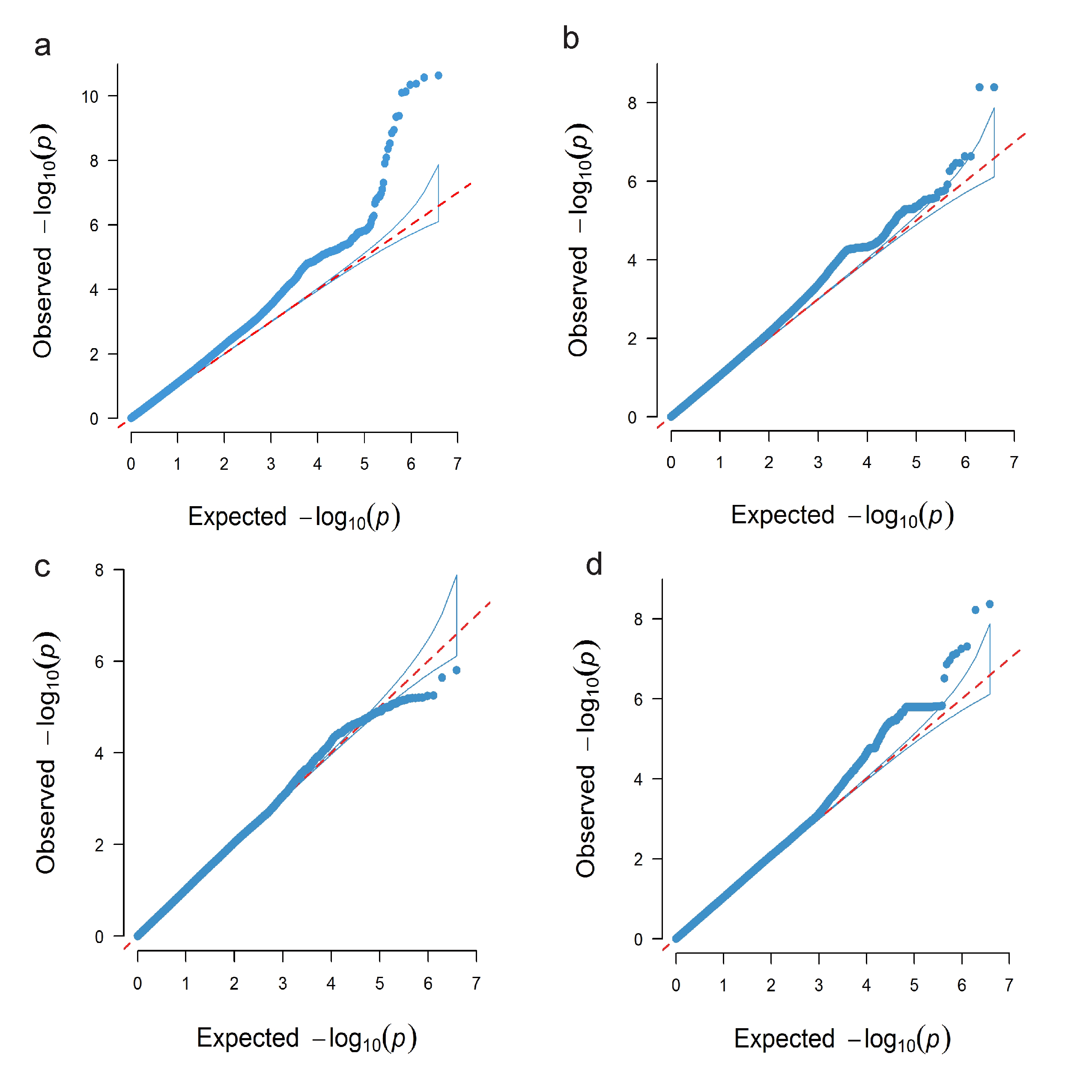


**Figure S5. The** [**Quantile-Quantile**](http://onlinestatbook.com/2/advanced_graphs/q-q_plots.html) **plots of our Han Chinese samples**. **a,** The meta-analysis of our ASA subgroup1, subgroup2 and GSA samples (3,493 cases and 4,709 controls, λ_GC_=1.104); **b,** The association result of ASA subgroup1 (2,055 cases and 1,823 controls, λ_GC_=1.065); **c,** The association result of ASA subgroup2 (607 cases and 1,186 controls, λ_GC_=1.015); **d,** The assocaiton result of GSA samples (831 cases and 1700 controls, λ_GC_=1.056).


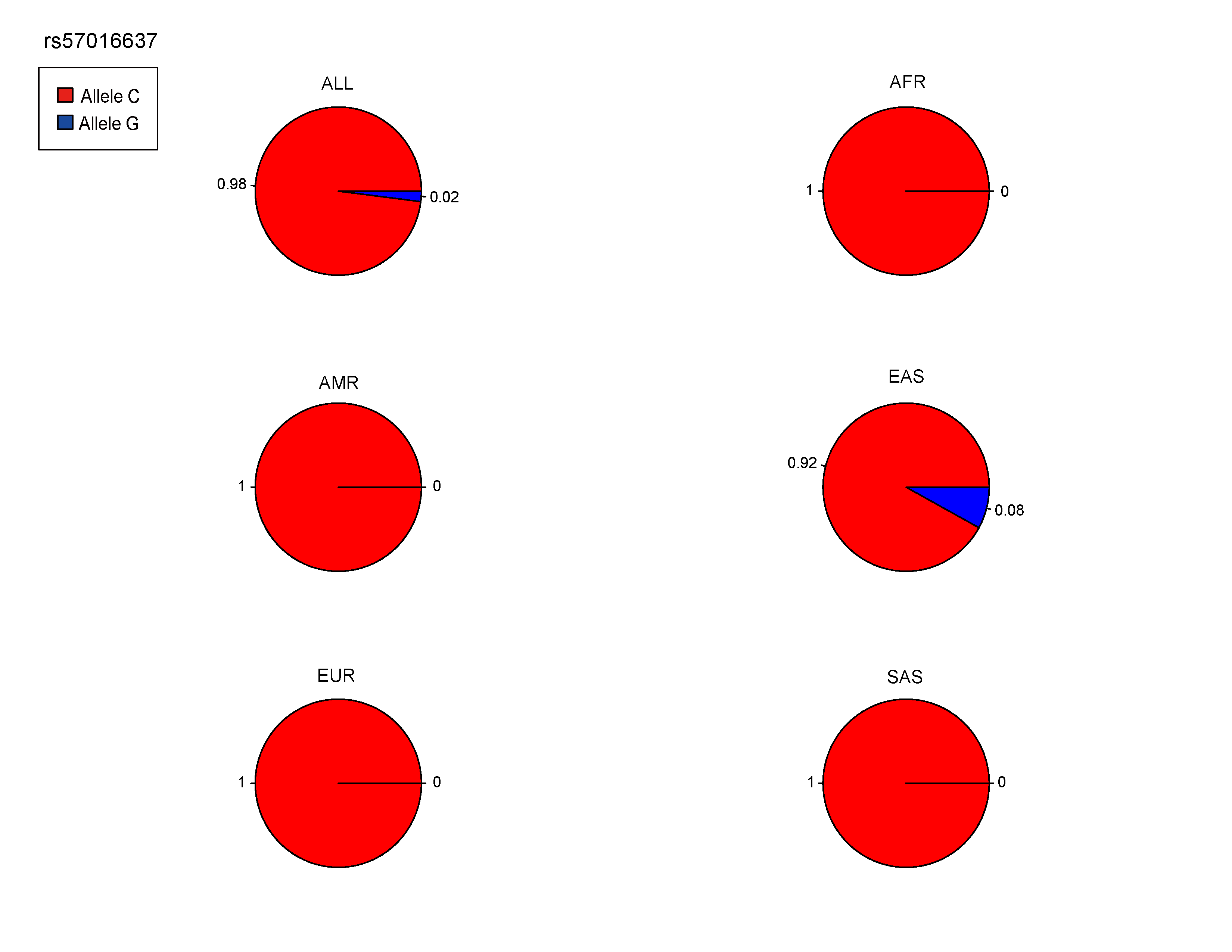


**Figure S6. The allelic frequency of rs57016637 in global populations from the 1000 Genome project**. ALL (all phase3 samples), AFR (African samples), AMR (American samples), EAS (East Asisan samples), EUR (European samples), SAS (South Asian samples).

**
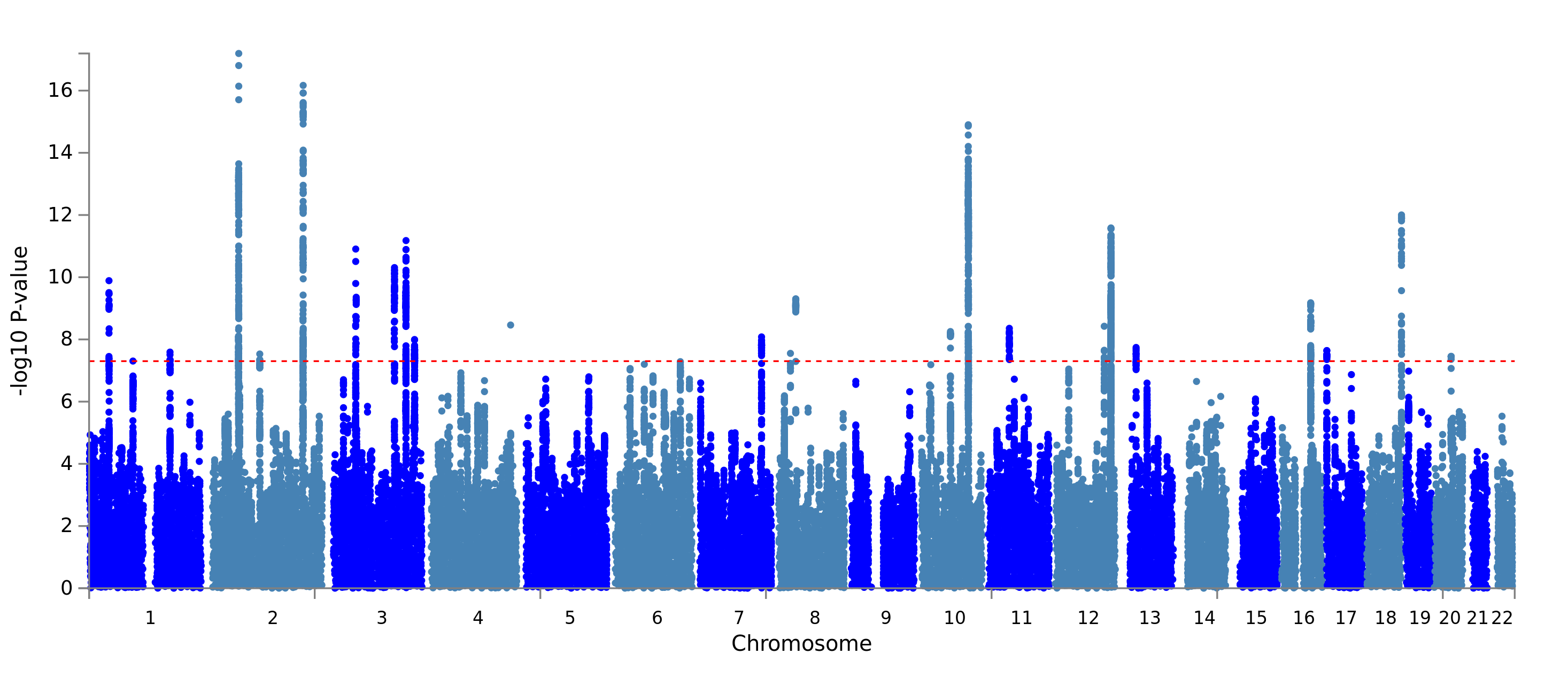
**

**Figure S7. The manhattan plot of meta-analysis result of our Han Chinese samples and East Asian samples (26,271 cases and 40,071 controls).**


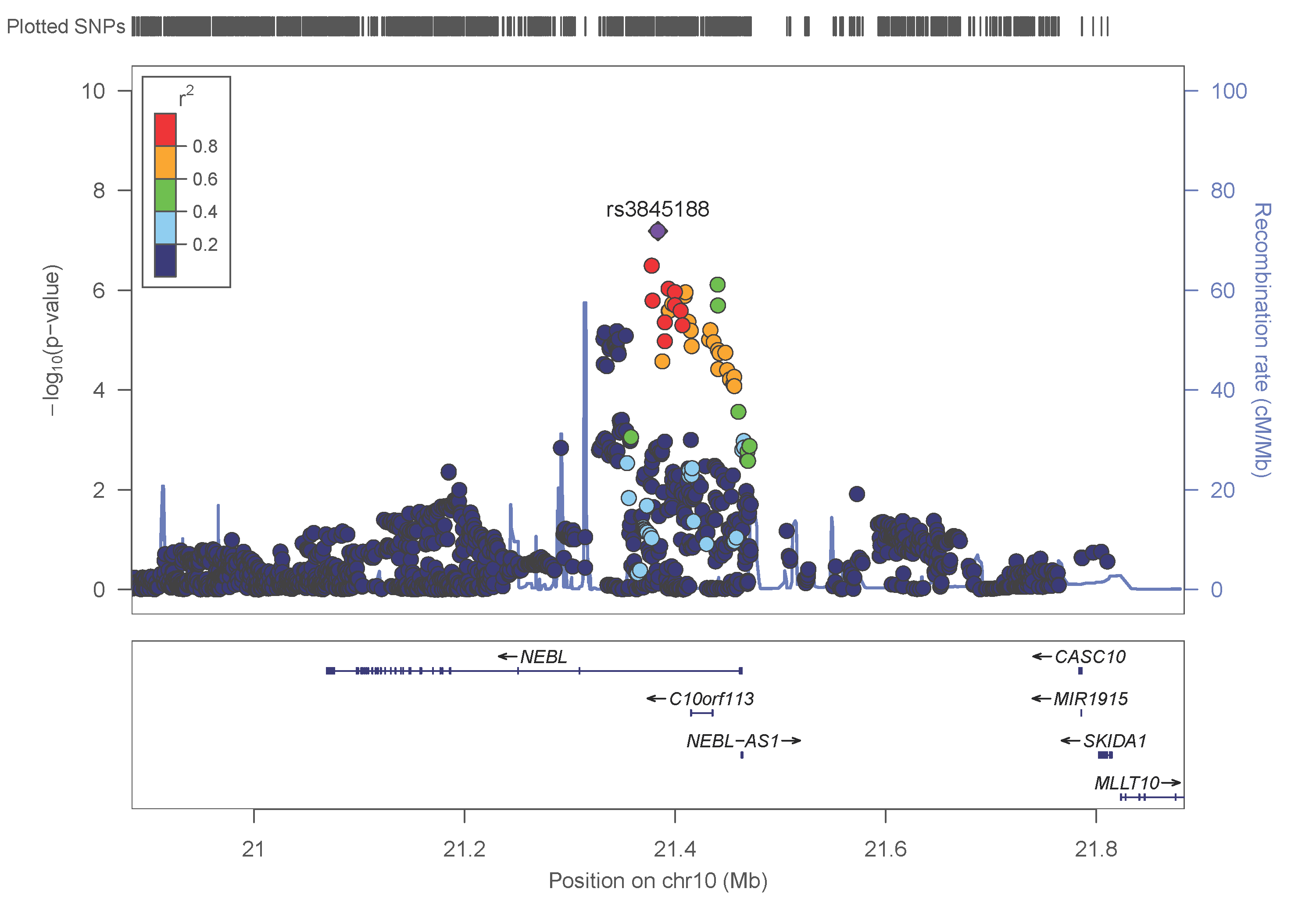


**Figure S8. The locuszoom plot of rs3845188 (*P*=6.50×10^-8^, OR=0.91).** The association result was from the combined EAS populations (including Han Chinese samples of our study and East Asian samples (26,271 cases and 40,071 controls)).


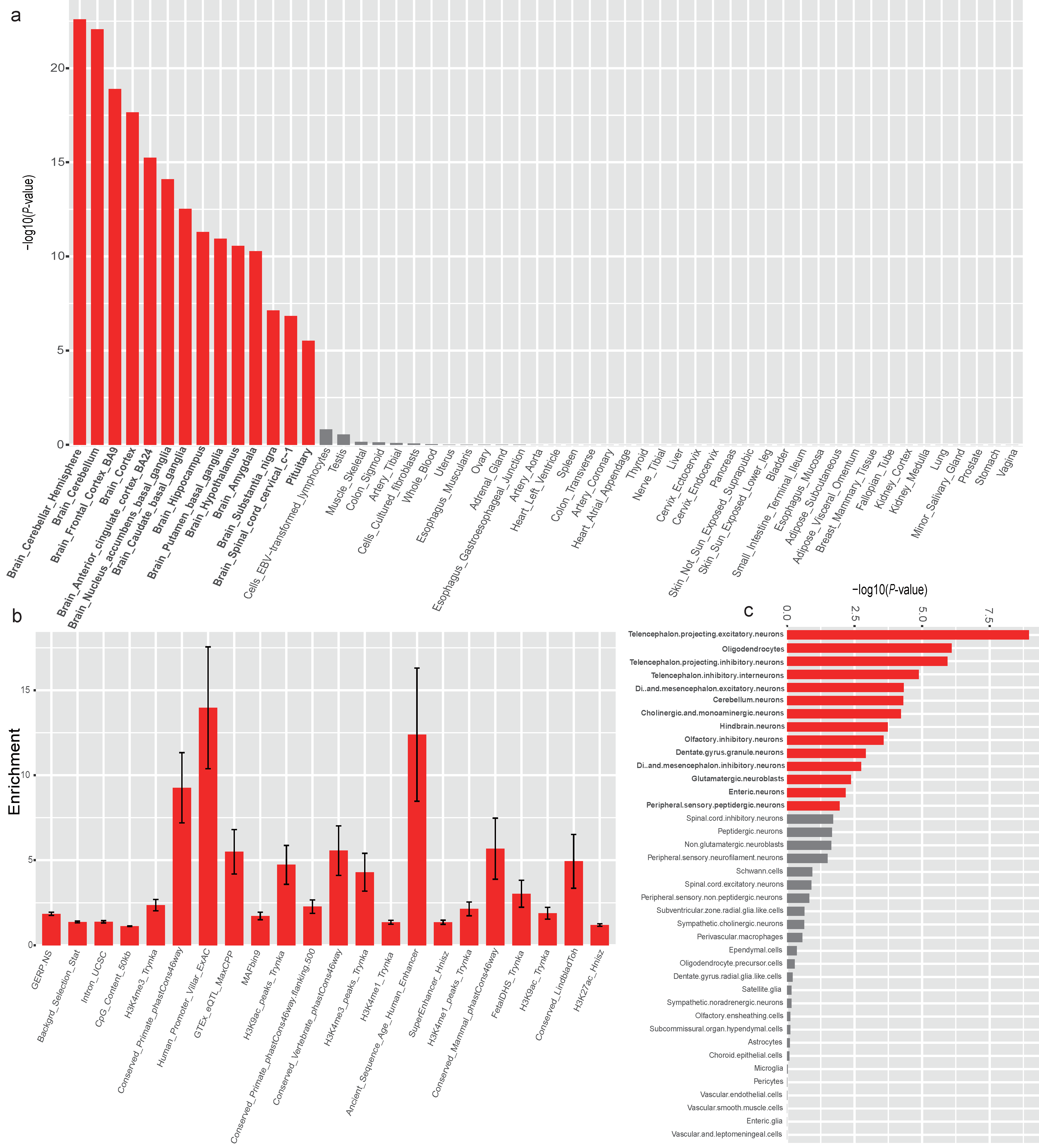


**Figure S9. Tissue and cell-type enrichment results.** **a,** Tissue enrichment results. Tissues that showed significant enrichment (FDR<0.05) were marked by red. **b**, LDSC heritability partitioning results. Functional annotations that showed significant enrichment (FDR<0.05) were marked by red. **c**, Cell-type enrichment analysis results. Cell types that showed significant enrichment (FDR < 0.05) were marked by red.

**Table S1. The detail association result of the new genome wide significant loci identified in this study in different meta-analysis datasets.**

| **Genomic Locus** | **Lead SNP** | **Chr** | **A1/A2** ^a^ | **Start** | **End** | **lab^b^** | | **Lab+EAS^c^** | | **Lab+EAS+EUR^d^** | |
| --- | --- | --- | --- | --- | --- | --- | --- | --- | --- | --- | --- |
|  |  |  |  |  |  | **P** | **OR** | **P** | **OR** | **P** | **OR** |
| 1 | rs7192086 | 16 | T/A | 13026440 | 13096582 | 4.92×10^-08^ | 1.22 | 7.34×10^-05^ | 1.07 | 9.58×10^-06^ | 1.05 |
| 2 | rs57016637 | 22 | G/C | 30745160 | 31426739 | 2.33×10^-11^ | 1.34 | 4.06×10^-04^ | 1.09 | NA^e^ | NA |
| 3 | rs115487049 | 2 | A/T | 76239791 | 76479233 | 0.32 | 1.09 | 0.089 | 1.07 | 4.72×10^-08^ | 1.07 |
| 4 | rs10178509 | 2 | T/C | 236769459 | 236835189 | 3.45×10^-04^ | 0.88 | 2.95×10^-06^ | 0.94 | 1.08×10^-08^ | 0.95 |
| 5 | rs1426271 | 3 | A/G | 188178427 | 188201380 | 0.099 | 0.94 | 4.71×10^-04^ | 0.95 | 3.23×10^-08^ | 0.95 |
| 6 | rs2911914 | 4 | T/G | 37799608 | 37853687 | 0.013 | 0.91 | 6.74×10^-07^ | 0.93 | 2.12×10^-08^ | 0.95 |
| 7 | rs6848123 | 4 | A/C | 80186915 | 80247342 | 0.0098 | 1.13 | 6.37×10^-06^ | 1.08 | 1.17×10^-08^ | 1.05 |
| 8 | rs319227 | 5 | A/C | 146229816 | 146326342 | 0.0047 | 1.11 | 4.12×10^-05^ | 1.06 | 6.11×10^-09^ | 1.05 |
| 9 | rs12202107 | 6 | T/C | 130544509 | 130768029 | 0.030 | 1.08 | 1.87×10^-05^ | 1.06 | 2.51×10^-08^ | 1.05 |
| 10 | rs9386072 | 6 | A/T | 144837796 | 144970207 | 6.46×10^-06^ | 0.86 | 5.19×10^-08^ | 0.92 | 1.01×10^-08^ | 0.93 |
| 11 | rs2106747 | 7 | A/G | 23529364 | 23922010 | 0.026 | 1.08 | 0.0031 | 1.04 | 3.36×10^-08^ | 1.05 |
| 12 | rs492430 | 7 | T/G | 100262123 | 100356544 | 0.14 | 1.11 | 0.0037 | 1.07 | 2.26×10^-08^ | 1.07 |
| 13 | rs59761926 | 9 | T/C | 10238145 | 10255606 | 0.0022 | 0.90 | 2.23×10^-07^ | 0.92 | 3.02×10^-09^ | 0.95 |
| 14 | rs7301566 | 12 | T/C | 50465355 | 50587778 | 0.14 | 1.09 | 3.83×10^-04^ | 1.09 | 2.91×10^-08^ | 1.06 |
| 15 | rs6563592 | 13 | T/G | 38769016 | 38825911 | 0.092 | 0.94 | 7.95×10^-05^ | 0.95 | 2.13×10^-08^ | 0.95 |
| 16 | rs2099108 | 14 | C/G | 81620883 | 81771563 | 9.81×10^-04^ | 1.12 | 2.58×10^-04^ | 1.06 | 2.67×10^-08^ | 1.05 |
| 17 | rs6100546 | 20 | T/C | 58247383 | 58253583 | 0.23 | 0.93 | 4.68×10^-04^ | 0.92 | 2.79×10^-08^ | 0.94 |

^a^ Odds ratio is based on A1; ^b^ GWAS result of our lab samples (3,493 cases, 4,709 controls); ^c^ GWAS meta-analysis result of our lab samples and East Asian samples (26,271 cases, 40,071 controls); ^d^ GWAS meta-analysis result of our lab samples and East Asian samples and PGC2 EUR samples (59,911 cases and 83,527 controls); ^e^ rs57016637 is not a polymorphism site in European population based on 1000 genome data.

**Table S2. Genes associated with the 17 newly identified lead SNPs in the human brain tissues**

| **SNP** | **Gene symbol** | | **Chr** | **Pvalue(cmc)** | **Pvalue(xQTL)** | **Pvalue(libd2)** | **Brain(libd2)** | **Pvalue(GTEx)** | **Brain(GTEx)** |
| --- | --- | --- | --- | --- | --- | --- | --- | --- | --- |
| rs12202107 | | *TMEM200A* | 6 | 1.47E-04 | NA | NA | NA | NA | NA |
| rs2099108 | | *TSHR* | 14 | 4.61E-06 | NA | NA | NA | NA | NA |
| rs2099108 | | *CEP128* | 14 | 9.81E-04 | NA | NA | NA | NA | NA |
| rs2099108 | | *STON2* | 14 | 4.80E-04 | 7.59E-03 | NA | NA | 1.60E-05 | Hippocampus |
| rs2106747 | | *FAM221A* | 7 | 1.47E-10 | 1.00E-22 | 9.58E-18 | HIPPO | 3.80E-08 | Spinal cord |
| rs2106747 | | *STK31* | 7 | NA | NA | 1.43E-10 | HIPPO | 3.10E-06 | Spinal cord |
| rs2911914 | | *PGM2* | 4 | 4.59E-11 | 1.02E-09 | 2.16E-07 | DLPFC | 1.40E-08 | Putamen |
| rs2911914 | | *TBC1D1* | 4 | NA | 1.23E-03 | NA | NA | NA | NA |
| rs2911914 | | *RP11-177C12.1* | 4 | NA | NA | NA | NA | 2.60E-05 | Caudate |
| rs319227 | | *PPP2R2B* | 5 | 8.61E-03 | NA | NA | NA | NA | NA |
| rs319227 | | *JAKMIP2* | 5 | NA | 1.07E-02 | NA | NA | NA | NA |
| rs492430 | | *GIGYF1* | 7 | 5.89E-03 | 6.12E-05 | NA | NA | 8.50E-06 | Substantia nigra |
| rs492430 | | *ZCWPW1* | 7 | 2.90E-04 | NA | NA | NA | NA | NA |
| rs492430 | | *GNB2* | 7 | NA | NA | 1.59E-04 | DLPFC | NA | NA |
| rs492430 | | *ACTL6B* | 7 | NA | 4.49E-04 | NA | NA | NA | NA |
| rs492430 | | *TRIP6* | 7 | NA | 9.25E-04 | NA | NA | NA | NA |
| rs492430 | | *AP1S1* | 7 | NA | 1.54E-02 | NA | NA | NA | NA |
| rs492430 | | *SLC12A9* | 7 | NA | 1.70E-02 | NA | NA | NA | NA |
| rs492430 | | *ACHE* | 7 | NA | 1.87E-02 | NA | NA | NA | NA |
| rs492430 | | *ZSCAN21* | 7 | NA | 3.01E-02 | NA | NA | NA | NA |
| rs492430 | | *PPP1R35* | 7 | NA | 4.27E-02 | NA | NA | NA | NA |
| rs492430 | | *PILRA* | 7 | NA | 4.97E-02 | NA | NA | NA | NA |
| rs492430 | | *TFR2* | 7 | NA | NA | NA | NA | 1.00E-05 | Nucleus accumbens |
| rs6100546 | | *TH1L* | 20 | NA | 2.34E-02 | NA | NA | NA | NA |
| rs6100546 | | *PHACTR3* | 20 | NA | 4.17E-02 | NA | NA | 1.30E-10 | Cerebellum |
| rs7192086 | | *ERCC4* | 16 | NA | 3.86E-02 | NA | NA | NA | NA |
| rs7301566 | | *COX14* | 12 | 1.74E-03 | 2.10E-02 | NA | NA | NA | NA |
| rs7301566 | | *DIP2B* | 12 | 5.48E-07 | 2.71E-03 | 2.84E-06 | DLPFC | 7.00E-05 | Putamen |
| rs7301566 | | *CERS5* | 12 | NA | 2.39E-13 | 1.18E-06 | HIPPO | 3.40E-06 | Putamen |
| rs7301566 | | *RP4-605O3.4* | 12 | NA | NA | 3.47E-09 | DLPFC | 8.70E-11 | Spinal cord |
| rs7301566 | | *SPATS2* | 12 | NA | 3.64E-03 | NA | NA | NA | NA |
| rs7301566 | | *ASIC1* | 12 | NA | 3.61E-02 | NA | NA | NA | NA |
| rs7301566 | | *ATF1* | 12 | NA | 3.93E-02 | NA | NA | NA | NA |

Uncorrected P values were showed.

**Table S3. Expression analysis of the potential eQTL target genes (of the newly identified lead SNPs) in schizophrenia cases and controls**

| Gene Name |  | Chr | Gene start | Gene stop | log2FC | P-value |
| --- | --- | --- | --- | --- | --- | --- |
| *STON2* |  | 14 | 81727000 | 81902809 | 0.190246713 | 2.27E-11 |
| *TSHR* |  | 14 | 81421333 | 81612646 | -0.244605649 | 1.67E-05 |
| *ASIC1* |  | 12 | 50451331 | 50477394 | -0.056796897 | 3.18E-05 |
| *GNB2* |  | 7 | 100271154 | 100276797 | 0.045785887 | 6.94E-05 |
| *PILRA* |  | 7 | 99965153 | 99997719 | -0.085394593 | 0.007108005 |
| *DIP2B* |  | 12 | 50898768 | 51142450 | -0.045081024 | 0.009929265 |
| *SLC12A9* |  | 7 | 100424442 | 100464631 | -0.034347456 | 0.014386702 |
| *ACTL6B* |  | 7 | 100240720 | 100254084 | 0.033532024 | 0.020440753 |
| *TRIP6* |  | 7 | 100464760 | 100471076 | 0.048146976 | 0.021313086 |
| *PHACTR3* |  | 20 | 58152564 | 58422766 | 0.023527186 | 0.032486931 |
| *ZCWPW1* |  | 7 | 99998449 | 100026615 | -0.040724551 | 0.035217281 |
| *CEP128* |  | 14 | 80943330 | 81425861 | 0.033495232 | 0.037233949 |
| *TMEM200A* |  | 6 | 130686879 | 130764208 | -0.052579775 | 0.045352486 |
| *PPP2R2B* |  | 5 | 145967936 | 146464347 | -0.01593939 | 0.045485911 |

**Expression data were from the PsychEncode(genes with an uncorrected P-value<0.05 were listed).**

**Table S4. The MAGMA gene set enrichment analysis result (items with FDR<0.10 were listed).**

| **FULL NAME** | **P** | **FDR** |
| --- | --- | --- |
| GO_NEURON_SPINE | 7.06E-07 | 0.0044 |
| GO_MEMBRANE_DEPOLARIZATION_DURING_ACTION_POTENTIAL | 4.72E-06 | 0.015 |
| GO_CYTOSOLIC_CALCIUM_ION_TRANSPORT | 4.03E-05 | 0.045 |
| GO_VOLTAGE_GATED_SODIUM_CHANNEL_COMPLEX | 4.09E-05 | 0.045 |
| GO_T_TUBULE | 4.32E-05 | 0.045 |
| GO_VOLTAGE_GATED_SODIUM_CHANNEL_ACTIVITY | 2.57E-05 | 0.045 |
| GO_VOLTAGE_GATED_ION_CHANNEL_ACTIVITY | 7.17E-05 | 0.063 |
| GO_CALCIUM_ION_TRANSMEMBRANE_IMPORT_INTO_CYTOSOL | 8.93E-05 | 0.069 |
| GO_REGULATION_OF_NEURONAL_SYNAPTIC_PLASTICITY | 0.00019 | 0.074 |
| GO_REGULATION_OF_SYNAPTIC_PLASTICITY | 0.00015 | 0.074 |
| GO_ENDOPLASMIC_RETICULUM_ORGANIZATION | 0.00018 | 0.074 |
| GO_NEURON_PROJECTION_TERMINUS | 0.00018 | 0.074 |
| GO_VOLTAGE_GATED_CATION_CHANNEL_ACTIVITY | 0.00016 | 0.074 |
| GO_SODIUM_CHANNEL_ACTIVITY | 0.00017 | 0.074 |
| GO_AV_NODE_CELL_TO_BUNDLE_OF_HIS_CELL_COMMUNICATION | 0.00020 | 0.074 |
| GO_NEUROTRANSMITTER_RECEPTOR_COMPLEX | 0.00020 | 0.074 |
| GO_INTRINSIC_COMPONENT_OF_SYNAPTIC_MEMBRANE | 0.00016 | 0.074 |
| GO_LEARNING | 0.00025 | 0.085 |
| GO_NMDA_SELECTIVE_GLUTAMATE_RECEPTOR_COMPLEX | 0.00032 | 0.098 |
| GO_RETINOID_X_RECEPTOR_BINDING | 0.00033 | 0.098 |
| GO_INTRINSIC_COMPONENT_OF_POSTSYNAPTIC_MEMBRANE | 0.00032 | 0.098 |

**Table S5. The TWAS result. Significant genes (after Bofferoni correction) were listed.**

| **Gene name** | **chr** | **TWAS.Z** | **TWAS.P** | **Repoted in PsychEncode** |
| --- | --- | --- | --- | --- |
| *C2orf47* | 2 | -10.4 | 1.57E-25 | yes |
| *MPHOSPH9* | 12 | -7.9402 | 2.02E-15 | yes |
| *DDAH2* | 6 | 7.5876 | 3.26E-14 | no |
| *ZNF184* | 6 | 7.2073 | 5.71E-13 | yes |
| *TYW5* | 2 | -7.20 | 6.25E-13 | yes |
| *SETD8* | 12 | -6.9918 | 2.71E-12 | yes |
| *ATF6B* | 6 | 6.8267 | 8.69E-12 | no |
| *NEK4* | 3 | 6.709732 | 1.95E-11 | no |
| *CEP170* | 1 | 6.580263 | 4.70E-11 | no |
| *NDUFA2* | 5 | -6.46662 | 1.00E-10 | no |
| *PCDHA2* | 5 | 6.37718 | 1.80E-10 | no |
| *CLCN3* | 4 | 6.33298 | 2.40E-10 | no |
| *SNAP91* | 6 | 6.2219 | 4.91E-10 | yes |
| *PTBP2* | 1 | 6.209449 | 5.32E-10 | no |
| *GLT8D1* | 3 | -6.199 | 5.68E-10 | no |
| *SLC45A1* | 1 | -5.985935 | 2.15E-09 | no |
| *THOC7* | 3 | -5.955598 | 2.59E-09 | yes |
| *LOC100507140* | 2 | -5.92 | 3.24E-09 | no |
| *TMEM110* | 3 | -5.861489 | 4.59E-09 | no |
| *ZMAT2* | 5 | 5.82354 | 5.76E-09 | yes |
| *HSPA1L* | 6 | -5.809 | 6.28E-09 | no |
| *USMG5* | 10 | 5.80789 | 6.33E-09 | no |
| *PCDHA7* | 5 | 5.76965 | 7.94E-09 | yes |
| *BTN3A3* | 6 | -5.7463 | 9.12E-09 | no |
| *ITIH4* | 3 | -5.704333 | 1.17E-08 | no |
| *CNTN4* | 3 | 5.661488 | 1.50E-08 | no |
| *MAN2A1* | 5 | 5.64978 | 1.61E-08 | no |
| *NT5C2* | 10 | -5.62708 | 1.83E-08 | no |
| *MED8* | 1 | -5.624045 | 1.87E-08 | no |
| *CUL9* | 6 | 5.5473 | 2.90E-08 | no |
| *FANCL* | 2 | -5.55 | 2.91E-08 | no |
| *CPNE7* | 16 | -5.4241 | 5.83E-08 | no |
| *SLC9B1* | 4 | -5.3623 | 8.22E-08 | no |
| *NEURL* | 10 | -5.35525 | 8.54E-08 | no |
| *LOC388152* | 15 | 5.3488 | 8.85E-08 | no |
| *TSHR* | 14 | -5.3045 | 1.13E-07 | no |
| *AKAP10* | 17 | 5.2551 | 1.48E-07 | no |
| *ZSCAN23* | 6 | -5.1666 | 2.38E-07 | yes |
| *MCHR1* | 22 | -5.1139 | 3.16E-07 | yes |
| *STON2* | 14 | 5.0875 | 3.63E-07 | no |
| *IK* | 5 | 5.07996 | 3.78E-07 | no |
| *SDCCAG8* | 1 | 5.053615 | 4.34E-07 | yes |
| *SFMBT1* | 3 | -4.902862 | 9.45E-07 | no |
| *NAGA* | 22 | 4.8981 | 9.68E-07 | yes |
| *RILPL1* | 12 | 4.8944 | 9.86E-07 | no |
| *XPNPEP3* | 22 | 4.8895 | 1.01E-06 | yes |
| *GATAD2A* | 19 | -4.8707 | 1.11E-06 | yes |
| *PCDHAC1* | 5 | 4.84274 | 1.28E-06 | no |
| *PCDHA8* | 5 | 4.80992 | 1.51E-06 | yes |
| *KIAA1908* | 7 | 4.794 | 1.63E-06 | no |
| *LY6H* | 8 | 4.76371 | 1.90E-06 | no |
| *ZNF165* | 6 | 4.7623 | 1.91E-06 | yes |
| *LIN28B* | 6 | 4.721 | 2.35E-06 | no |
| *NIP7* | 16 | -4.68 | 2.86E-06 | yes |
| *PGBD1* | 6 | 4.6782 | 2.89E-06 | no |
| *FAM109B* | 22 | 4.6446 | 3.41E-06 | yes |
| *COG8* | 16 | 4.6242 | 3.76E-06 | yes |
| *RNF111* | 15 | -4.5965 | 4.30E-06 | no |
| *CPEB1* | 15 | -4.5921 | 4.39E-06 | no |
| *PCDHA13* | 5 | -4.56689 | 4.95E-06 | no |
| *TMEM81* | 1 | 4.565934 | 4.97E-06 | no |
| *SATB2* | 2 | -4.54 | 5.75E-06 | no |
| *TAP2* | 6 | -4.5274 | 5.97E-06 | no |
| *PDF* | 16 | -4.5178 | 6.25E-06 | yes |
| *ELOVL7* | 5 | 4.5157 | 6.31E-06 | no |
| *PPP1R13B* | 14 | -4.5144 | 6.35E-06 | yes |
| *VPS37A* | 8 | -4.5131 | 6.39E-06 | no |
| *RCBTB1* | 13 | -4.5124 | 6.41E-06 | no |
| *ZKSCAN3* | 6 | 4.4975 | 6.87E-06 | yes |
| *C5orf63* | 5 | 4.47008 | 7.82E-06 | no |
| *ELOVL1* | 1 | 4.463537 | 8.06E-06 | no |
| *ACADVL* | 17 | -4.4154 | 1.01E-05 | no |
| *MED30* | 8 | -4.40634 | 1.05E-05 | no |
| *HCG4* | 6 | 4.4035 | 1.07E-05 | no |
| *HIST1H4H* | 6 | -4.3799 | 1.19E-05 | yes |
| *WDR55* | 5 | 4.34557 | 1.39E-05 | no |
